# Fibrous dysplasia for radiologists: beyond ground glass bone matrix

**DOI:** 10.1007/s13244-018-0666-6

**Published:** 2018-11-27

**Authors:** Yevgeniya S. Kushchayeva, Sergiy V. Kushchayev, Tetiana Y. Glushko, Sri Harsha Tella, Oleg M. Teytelboym, Michael T. Collins, Alison M. Boyce

**Affiliations:** 1 Diabetes, Endocrinology, and Obesity Branch, National Institute of Diabetes and Digestive and Kidney Diseases, National Institutes of Health, 31 Center Dr, Bethesda, MD 20892 USA; 2 Division of Neuroradiology, Department of Radiology, Johns Hopkins Hospital, 1800 Orleans St, Baltimore, MD 21287 USA; 3 Department of Radiology, Mercy Catholic Medical Center, 1500 Lansdowne Ave, Darby, PA 19050 USA; 4 Department of Medicine, Division of Endocrinology, Diabetes and Metabolism, University of South Carolina School of Medicine, 6311 Garners Ferry Rd, Columbia, SC 29209 USA; 5 Skeletal Disorders and Mineral Homeostasis Section, National Institute of Dental and Craniofacial Research, National Institutes of Health, 30 Convent Drive Room 228 MSC 4320, Bethesda, MD 20892 USA

**Keywords:** Fibrous dysplasia, McCune-Albright syndrome, Ground glass bone matrix, Mazabraud’s syndrome, Skeletal radiology

## Abstract

**Abstract:**

Fibrous dysplasia (FD) is a congenital disorder arising from sporadic mutation of the α-subunit of the Gs stimulatory protein. Osseous changes are characterised by the replacement and distortion of normal bone with poorly organised, structurally unsound, fibrous tissue. The disease process may be localised to a single or multiple bones. In McCune-Albright syndrome (MAS), fibrous dysplasia is associated with hyperfunction of endocrine organs and overproduction of melanin in the skin, while Mazabraud syndrome FD is associated with intramuscular myxomas. In radiology, FD is very often automatically associated with the term “ground glass matrix”. However, FD is a complex disease, and knowledge of its unique pathogenesis and course are crucial to understanding imaging findings and potential complications. This article aims to not only summarise the spectrum of radiological findings of osseous and extra-osseous abnormalities associated with FD but also to highlight the pathological base of the disease evolution, corresponding imaging changes and complications based on the disease distribution. We also have provided current recommendations for clinical management and follow-up of patients with FD.

**Teaching Points:**

• *FD is often a part of complex disease, involving not only bone but also multiple other organs.*

• *FD lesions are characterised by age-related histological, radiographical and clinical transformations.*

• *Radiologists play a crucial role in the identification of osseous complications associated with FD.*

• *The craniofacial form of the disease is the most common type of FD and the most difficult form to manage.*

• *Patients with McCune-Albright syndrome may have different extra-skeletal abnormalities, which often require follow-up.*

## Introduction

Fibrous dysplasia (FD) is a disorder caused by sporadic mutation of the α-subunit of the Gs stimulatory protein, in which bone is replaced and distorted by poorly organised, structurally unsound, fibrous tissue. In McCune-Albright and Mazabraud syndromes, FD is associated with a range of extra-skeletal abnormalities. For many radiologists, FD is often automatically associated with the term “ground glass bone matrix”; however, FD is often a part of complex disease, and knowledge of its unique pathogenesis and disease course is crucial to understanding imaging findings, their changes over time, and potential complications.

This article summarises more than 25 years of observation of patients with FD under the protocol “Screening and natural history study of fibrous dysplasia” at the National Institutes of Health (Bethesda, Maryland, USA), (SNHFD, protocol 98-D-0145). We describe the spectrum of osseous and extra-osseous radiological findings related to FD and highlight the pathological base of disease evolution, corresponding imaging changes, complications based on disease distribution, and important surveillance and management techniques for radiologists.

## Nomenclature

The disease process may be localised to a single bone (monostotic FD) or multiple bones (polyostotic FD). The current convention is to preserve the eponym Jaffe-Lichtenstein disease for isolated FD. The name McCune-Albright syndrome is used when FD is associated with extra-skeletal abnormalities. Mazabraud syndrome is reserved for cases of FD with associated intramuscular myxomas. Cherubism has historically been considered a variant of FD, but it is a genetically distinct disease. This format has been codified in the Paris Nomenclature of Constitutional Diseases of the Bone [1] (Table 1).

**Table 1:** Nomenclature of the diseases associated with fibrous dysplasia (FD) lesions

Forms of fibrous dysplasia	Bone involvement		Café-au-lait spots	Endocrine disorders	Intramuscular myxomas
	Single	Multiple			
Monostotic	X				
Polyostotic		X			
McCune-Albright syndrome		X	X	X	
Mazabraud syndrome		X			X

## Pathogenesis and pathology

**Fig. 1:**
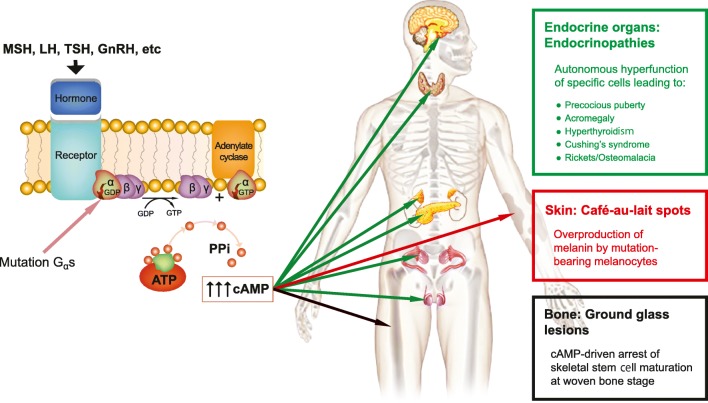
Post-zygotic mutations of the α-subunit of the Gs stimulatory protein (*GNAS* mutations) lead to the inappropriate production of the cyclic adenosine monophosphate (cAMP). In skin, the increased concentration of cAMP results in overproduction of the enzyme tyrosinase and abnormally high melanin production, incomplete differentiation of marrow stromal cells to abnormal osteoblasts with abnormal maturation of the bony matrix, and hyperfunction of the endocrine organs

**Fig. 2:**
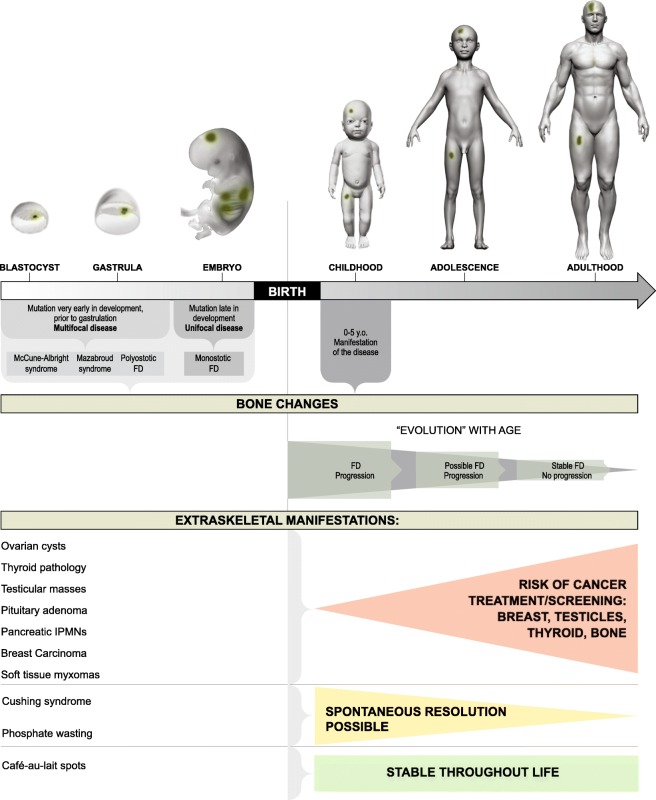
Mutation timing determines the extent of the disease and clinical manifestations. The stage of embryogenesis during which a mutation occurs, and the locations to where mutated progenitors subsequently migrate, determines if a patient will have a single lesion, polyostotic disease or one of the FD-related syndromes. Mutations that occur at early stages of embryogenesis result in the widespread distribution of the lesions. Mutations that develop at late stages of embryogenesis lead to more focused distribution of the lesions. Patients with McCune-Albright syndrome (MAS) may have different extra-skeletal abnormalities. Some of these abnormalities may progress to malignancy; part of them become stable throughout life; some abnormalities can regress or disappear

**Fig. 3:**
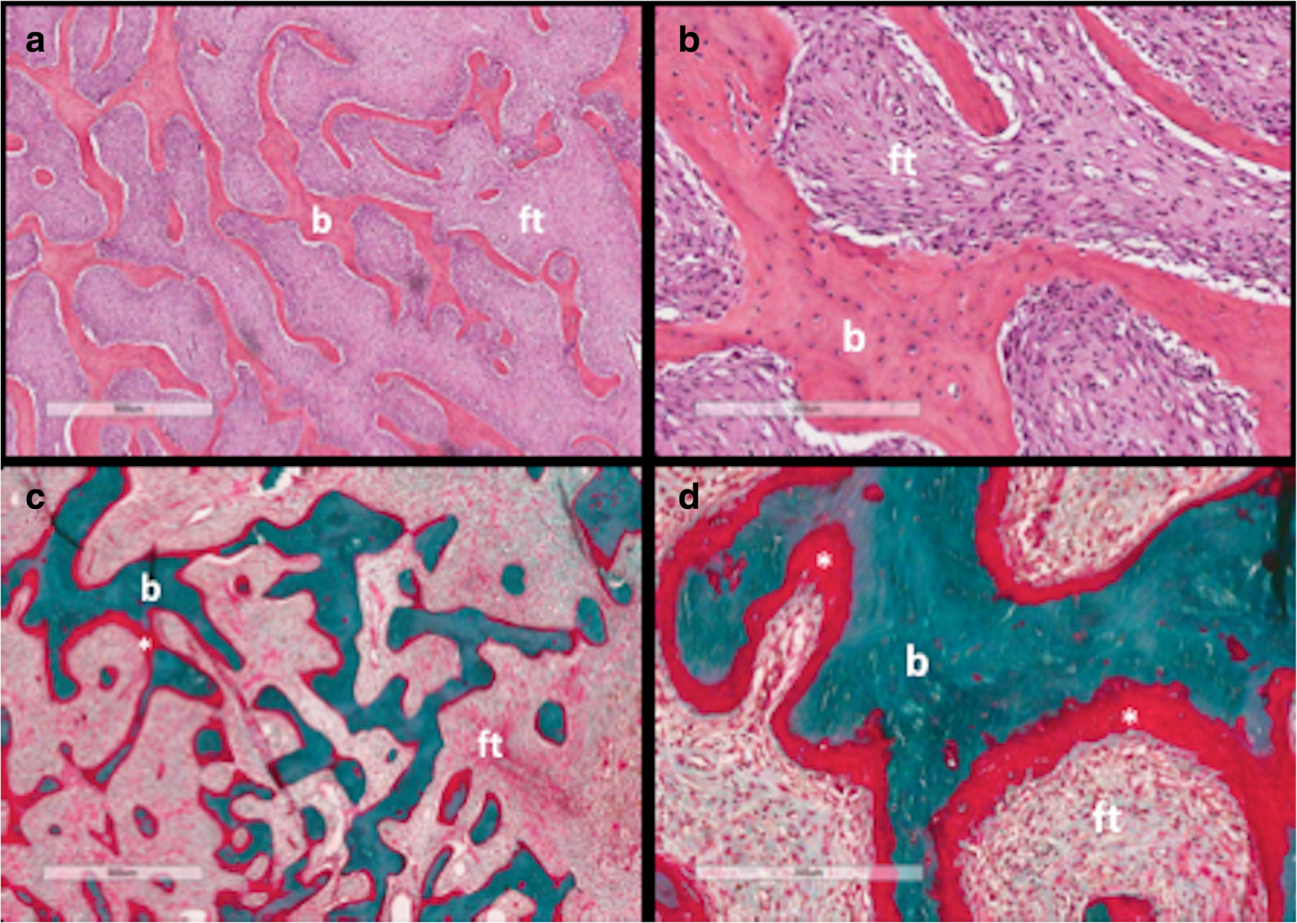
Histopathological features of fibrous dysplasia (FD). FD lesions are composed of fibrous tissue interspersed between bone trabeculae. The amount of bone within lesions is quite variable. Trabeculae are dysplastic, non-stress oriented, and appear disorganised. Haematoxylin-eosin stained sections in low (**a**) and high power (**b**) show irregular, discontinuous trabeculae (*b*) within a fibrous stroma (*ft*), demonstrating the typical “alphabet soup” pattern. Goldner’s trichrome stained sections in low (**c**) and high power (**d**) reveal osteomalacic changes including excess osteoid (*asterisks*) and severe undermineralisation of the dysplastic bone (reprinted from Boyce [34])

FD arises sporadically, and there are no confirmed cases of vertical transmission. Morphological changes in FD are related to post-zygotic mutations of the α-subunit of the G_s_ stimulatory protein (*GNAS* mutations) leading to activation and inappropriate cyclic adenosine monophosphate (cAMP) overproduction (Figs. 1 and 2) [2]. The monostotic form never progresses to polyostotic FD or McCune-Albright syndrome (MAS), and spontaneous resolution of FD does not occur. In bones, the mutation is responsible for creating bone marrow stromal cells with an impaired capacity to differentiate towards mature osteoblasts, adipocytes, and haematopoietic cells—supporting stroma, resulting in stroma devoid of haematopoietic marrow (Fig. 3) [3]. Haemorrhage and cystic changes may occasionally be seen, which may have overt secondary changes resembling an aneurysmal bone cyst (ABC). FD most commonly behaves as a slow and indolent growing mass lesion. The FD lesions may be described as quiescent (stable with no growth), non-aggressive (slow growing), or aggressive (rapid growth and may be associated with pain, paraesthesia, pathological fracture, malignant transformation) [4]. The activating *GNAS* mutations result in hyperfunctioning endocrine organ changes and melanin overproduction in skin. The vast majority of extra-skeletal abnormalities exist throughout life, with the exception of Cushing’s syndrome and phosphaturia [3].

## Monostotic and polyostotic fibrous dysplasia

### Imaging characteristics

The distribution of FD lesions depends on the form of the disease. Monostotic FD accounts for about 80% of patients with FD. The most common location of monostotic FD is the rib, skull and femur. In the polyostotic form, the skull, mandible, pelvic bones and femur are the most frequently affected sites. Many cases of monostotic FD are discovered incidentally, while the polyostotic form is usually diagnosed during the first few years of life, and the majority of bony lesions become non-silent and clinically significant by age 10 years, with almost no new lesions appearing after the age of 15. There is no difference in appearance of bony lesions between FD, MAS and Mazabraud syndrome, but syndromic patients typically have polyostotic disease.

FD lesions are not static morphological abnormalities. They are characterised by age-related histological, radiological and clinical transformations. In early childhood, lesions are metabolically active, and expand during linear growth. The lesions typically become static in size after puberty, and metabolic activity may decrease throughout adulthood [5].

#### Radiographs

**Fig. 4:**
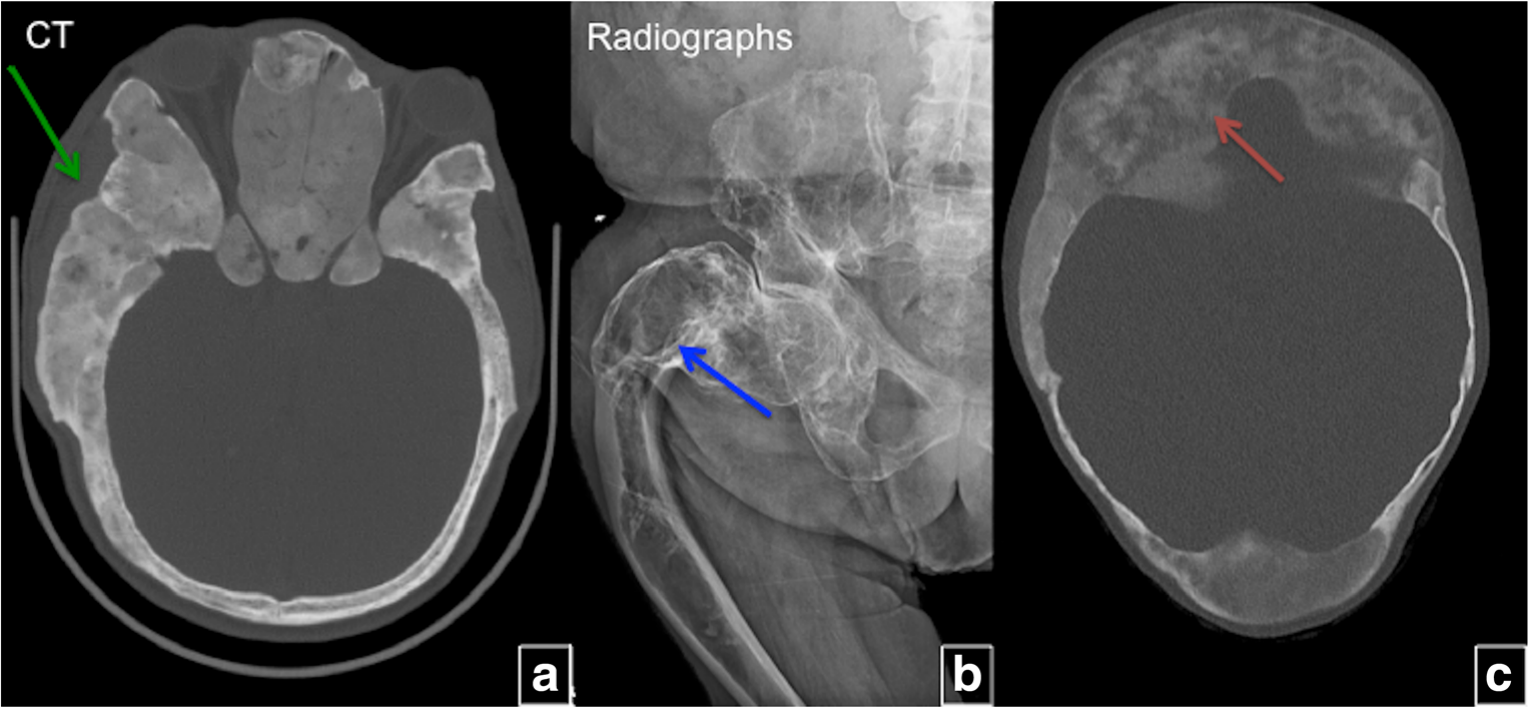
The location-based difference in appearance of fibrous dysplasia (FD) lesions. **a** Craniofacial FD demonstrates dense, sclerotic lesions (*green arrow*). **b** Lesions in the long bones and axial skeleton are typically lucent. There is a typical lesion in the proximal femur with characteristic lucent ground glass appearance and shepherd’s crook deformity (*blue arrow*). **c** Mixed radiolucent/lytic FD lesions in the skull

**Fig. 5:**
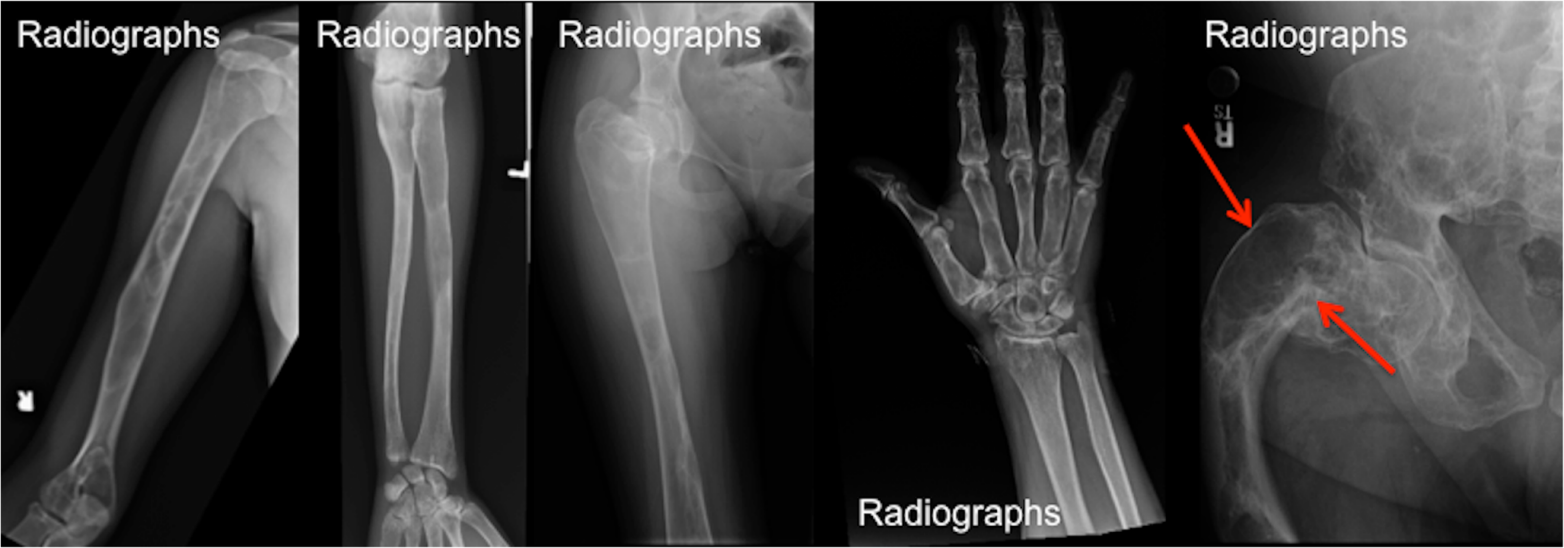
The radiographic appearance of fibrous dysplasia (FD) and the rind sign. **a**–**e** Frontal radiographs demonstrate classic FD lesions in appendicular skeleton. A classic lucent lesion surrounded by a layer of sclerotic reactive bone (so-called the rind sign). The rind sign is most commonly seen in the proximal femur (*red arrow*)

The spectrum of bone lesions can be classified into three primary bony patterns: cystic, sclerotic and mixed. A typical FD lesion in the axial skeleton appear as an area of radiolucent ground glass matrix, which is usually smooth and homogeneous, not centrally located within medullary bone. Craniofacial FD typically demonstrates dense and sclerotic lesions (Fig. 4). FD lesions can vary in size from a small, focal abnormality to a large lesion, perhaps involving most or all of a long bone (Fig. 5). Delicate fine trabeculae can be seen within FD lesions. The lesions usually cause cortical thinning due to enlarged fibro-osseous masses within the bone. The periosteal reaction is not usually present unless it is associated with a pathological fracture. Although endosteal scalloping may be present, a smooth outer cortical contour is always maintained. The lesion may undergo expansile remodelling secondary to the enlarging mass of fibro-osseous tissue. A thick layer of sclerotic bone is known as a rind sign (Fig. 5e). The sclerotic margins can vary in thickness and may be interrupted or incomplete. Small islands of cartilage, which later ossify and are seen as dense punctate or flocculent calcifications within FD lesions, can also be seen. This combination of enchondromata within an FD lesion, referred to as fibro cartilaginous dysplasia, is most frequently seen in the proximal femur.

#### Computed tomography (CT)

**Fig. 6:**
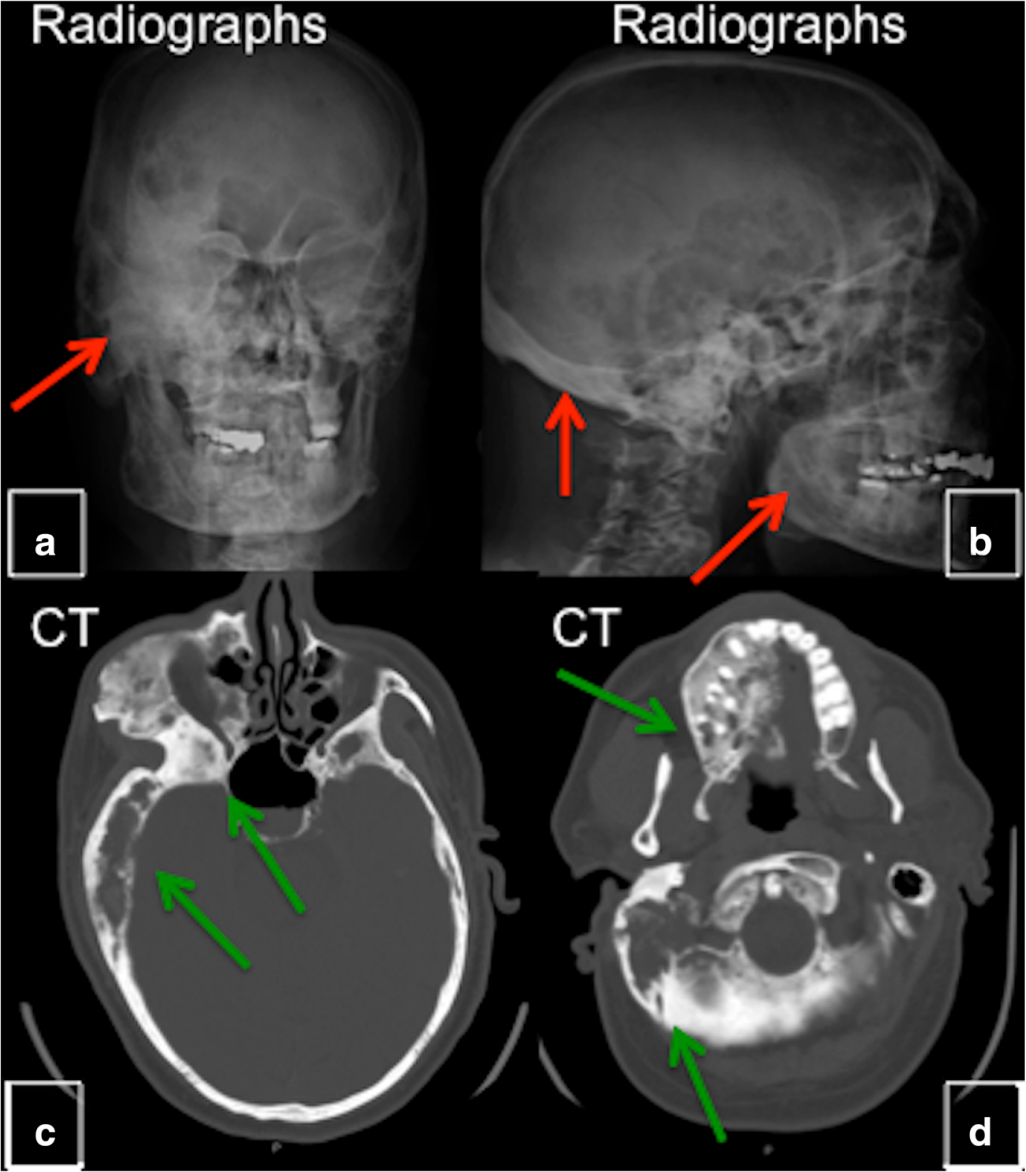
CT in fibrous dysplasia (FD). CT imaging is the modality of choice and superior to radiographs in delineating morphological changes in bone. Radiographs are not recommended for diagnostic purposes or for the characterisation of craniofacial lesions. **a**, **b** Radiographs of the head show evidence of FD (*red arrow*). **c**, **d** CT of the head on the same patient delineates lesions and demonstrates relationship between lesions and neuronal, vascular and soft tissue structures (*green arrow*)

CT imaging, which better delineates morphological changes in bone, is the modality of choice and superior to radiographs [4]. CT defines the anatomy of individual lesions and establishes the extent of disease. Plain radiographs are not recommended for diagnostic purposes and characterisation of craniofacial lesions; while CT and/or magnetic resonance tomography (MRI) evaluation of long bones is rarely indicated [6] (Fig. 6). Usually, attenuation of FD lesions ranges from 60 to 140 HU. CT scans may identify soft tissue masses, bone destruction and suggest malignant transformation. Lesions usually are enhanced after intravenous contrast administration.

#### MRI

**Fig. 7:**
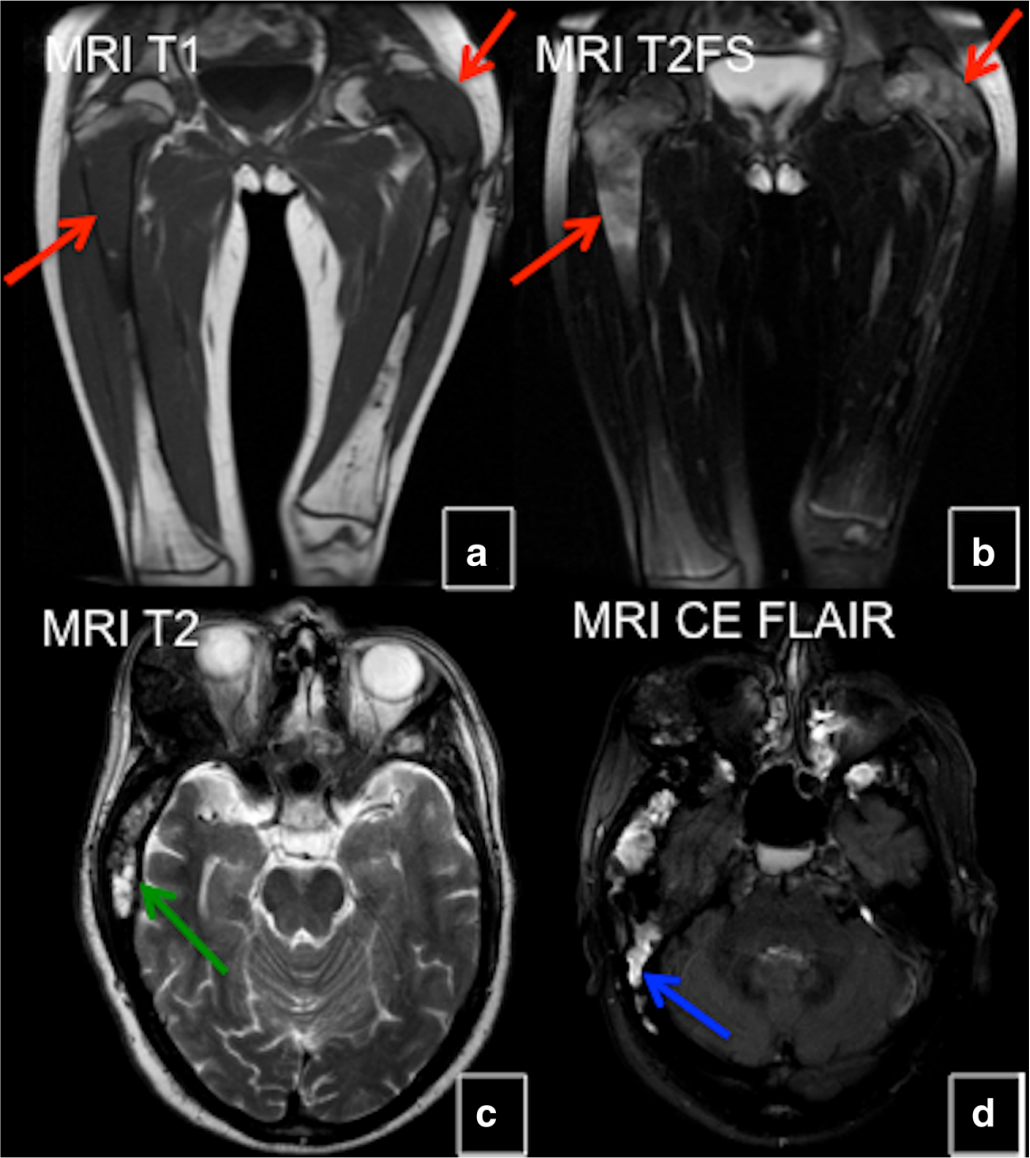
MRI in fibrous dysplasia (FD). **a**, **b** MRI typically shows sharply demarcated lesions with intermediate to low signal intensity on T1-weighted images (WI) and intermediate to high on T2-WI (*red arrow*). **c** Some FD lesions may also contain small cystic areas, which make the T2 signal bright (*green arrow*). **d** FD lesions usually show some degree of enhancement after contrast administration (*blue arrow*)

**Fig. 8:**
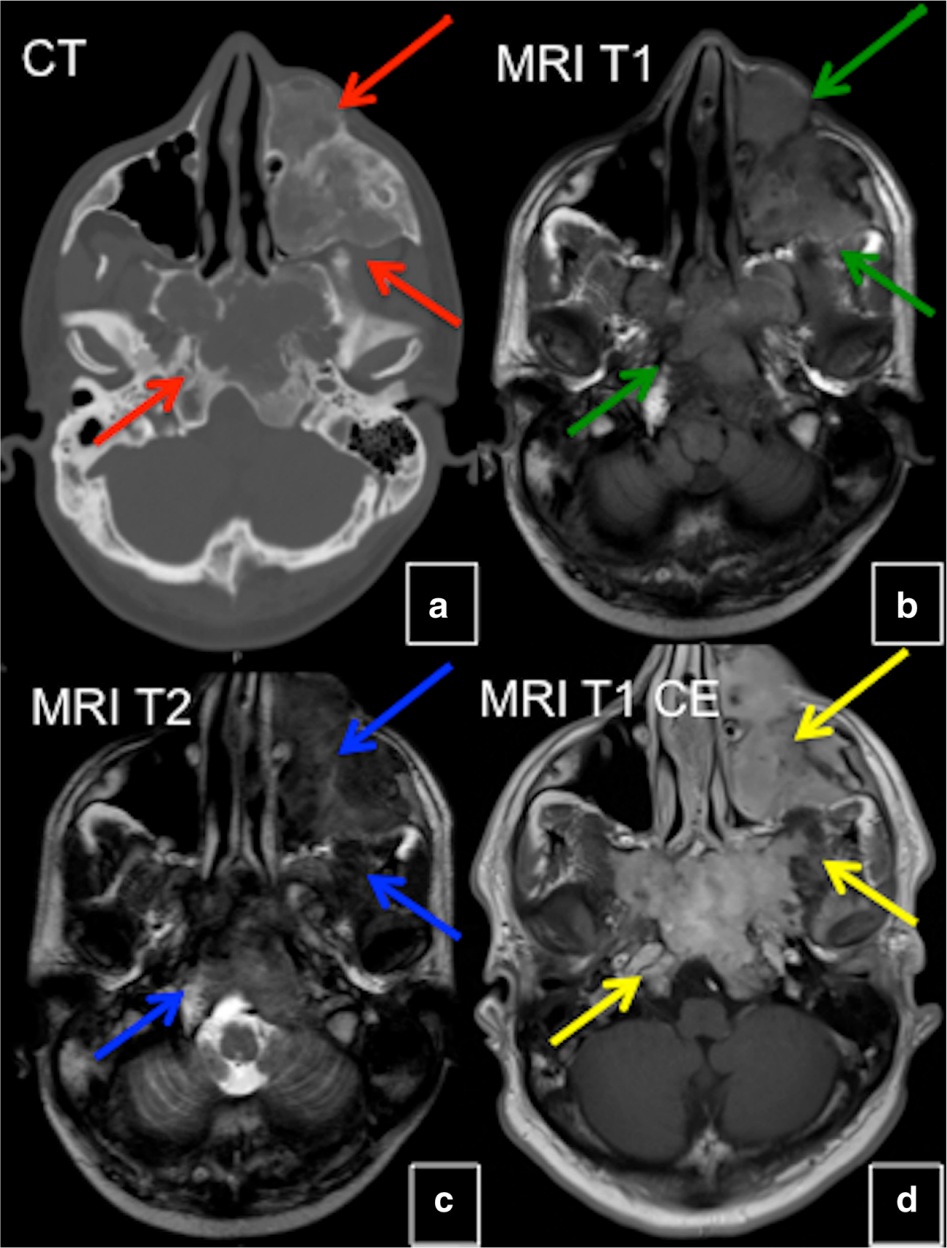
CT and MRI in craniofacial fibrous dysplasia (FD). **a** CT demonstrates mixed sclerotic FD lesions involving the skull base (*red arrows*). **b** Lesions demonstrate intermediate signal intensity on T1 weighted MRI (*green arrows*). On T2 weighted images lesions demonstrate heterogeneous hypointense/intermediate signal intensity (*blue arrows*). **d** FD lesions show slightly heterogeneous enhancement (*yellow arrows*)

**Fig. 9:**
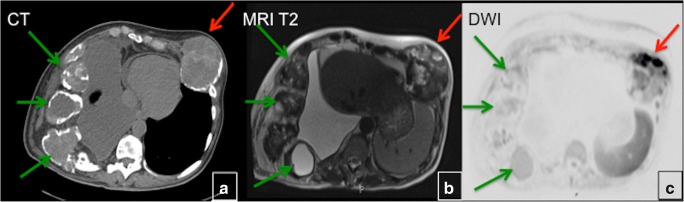
The significance of MRI with diffusion-weighted imaging (DWI) in the patient with polyostotic fibrous dysplasia (FD). CT (**a**), T2-weighted MRI (**b**) and DWI (**c**) show multiple rib lesions (*green arrows*). The left rib lesion (*red arrow*) demonstrates restricted diffusion, which requires further evaluation to rule out malignant transformation

FD does not consistently show hypointense signal intensities on T1- and T2-weighted images (WI) as might be expected [7]. Signal intensity on T1- and T2-WI and the degree of contrast enhancement depend on the amount and degree of bony trabeculae, cellularity, collagen, and cystic and haemorrhagic changes. Typically, FD lesions show sharply demarcated borders and intermediate to low signal intensity on T1-WI and intermediate to high intensity on T2-WI (Figs. 7 and 8). The higher the number of bony trabeculae, the lower the T2 signal, and vice versa—the fewer bony trabeculae, the higher T2 signal. FD lesions may also contain small cystic areas, which make the T2 signal brighter. All lesions showed some degree of enhancement on post-contrast T1-WI. Active lesions show avid enhancement, while inactive lesions show milder enhancement [8]. The enhancement pattern may be patchy central, rim, homogeneous or a combination. Therefore, MRI is not particularly useful in differentiating FD from other entities. This technique is helpful for the evaluation of complex cases of FD, such as in patients with compression of neurological structures in the brain and spinal canal. The technique may be useful in assessing malignant change and demonstrating the extension of tumour into the surrounding soft tissues. Diffusion-weighted imaging (DWI) may be helpful in differentiating benign from malignant osseous lesions, especially in the skull [9]. ADC values in skull lesions correlate with cell density and can potentially narrow the differential diagnoses for indeterminate lesions (Fig. 9). MRI is the modality of choice for suspected ABCs in FD lesions.

#### Nuclear medicine scans

**Fig. 10:**
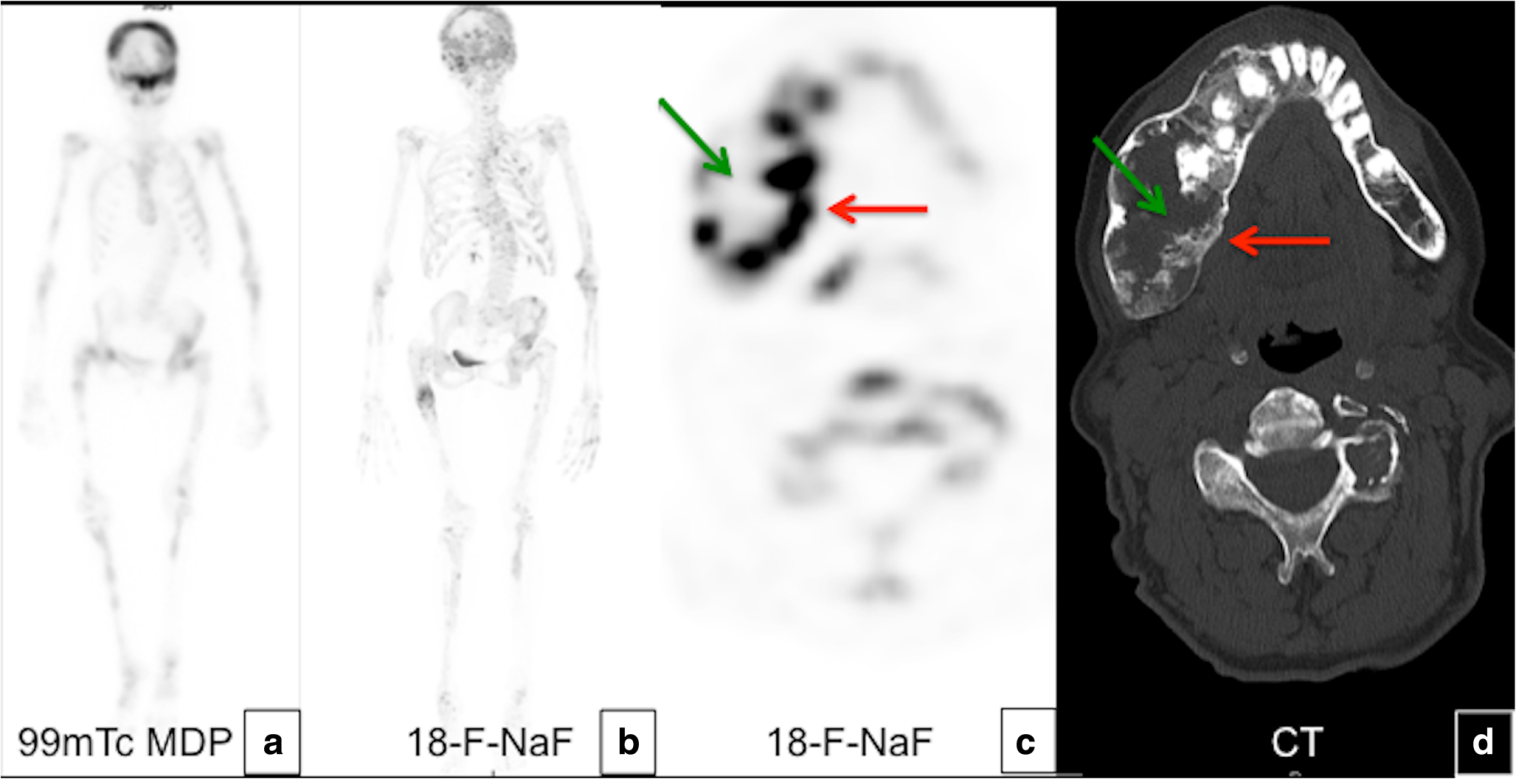
Nuclear medicine imaging in fibrous dysplasia (FD). **a** Bone scans with 99m-Tc-MDP are exquisitely sensitive at detecting the presence and extend of the disease. **b** 18-F-NaF on the same patient with polyostotic FD demonstrates multiple areas of focal radiotracer uptake corresponding to FD lesions. **c**, **d** 18-F-NaF PET/CT shows heterogeneous uptake by the lesions in the jaw and the spine with a central photopenic area (*green arrow*) and a peripheral metabolically active area (*red arrow*)

**Fig. 11:**
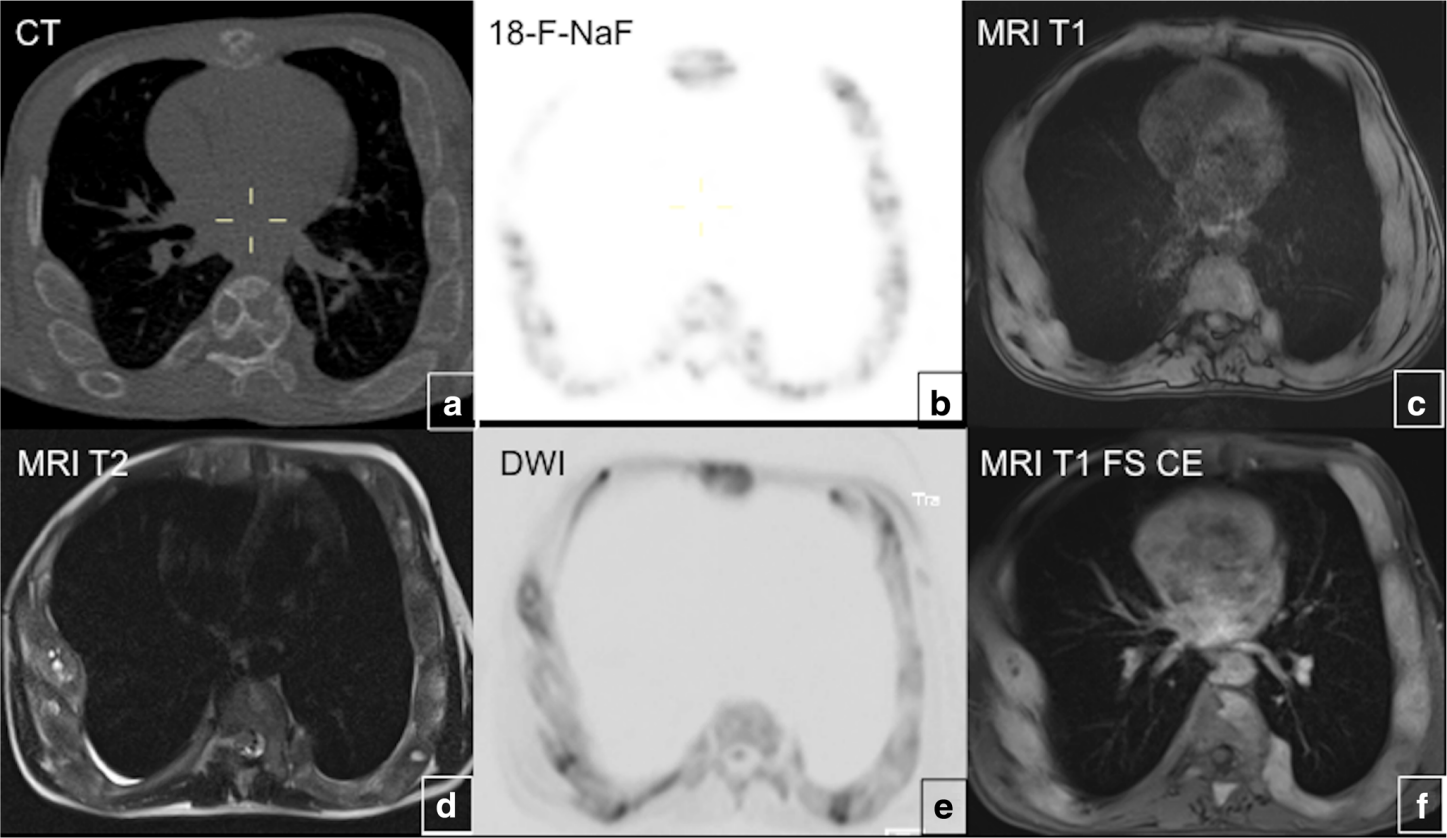
Multimodality imaging in polyostotic fibrous dysplasia (FD). **a**, **b** PET/CT with 18-F-NaF demonstrates multiple lucent FD lesions seen on CT with corresponding areas of mild radiotracer uptake on PET. **c**–**f** Lesions demonstrate intermediate T1 signal intensity on MRI (**c**), intermediate-to-low signal intensity on T2 (**d**), slightly hyperintense signal intensity on DWI (**e**), uniform enhancement after contrast administration (**f**)

Technetium 99m-methyldiphosphonate (99m-Tc-MDP) bone scan is used to detect metabolically active lesions and assess the extent of disease, especially in young patients [10]. Small areas of involvement may escape detection by the initial bone scan if the child is younger than 6 years old. Radiographs are used selectively to monitor the progression of the lesions initially identified on bone scans. After an initial diagnostic bone scans, a follow-up bone scan is not recommended. FD lesions show various 18-F-fluorodeoxyglucose (18-F-FDG) and 18-F-sodium fluoride (18-F-NaF) uptakes on PET/CT and may mimic malignant lesions or metastases (Figs. 10 and 11). Uptake of 18-F-FDG in FD may change with time. PET/CT cannot be used to identify areas of malignant transformation; however, rapid increased 18-F-FDG uptake may suggest the possibility of sarcomatous change. FD lesions were found to be positive on In-111 pentreotide (Octreoscan), Ga-67 citrate and Tc-99m MIBI scintigraphy as well as on 68-Ga-DOTATATE and 11-C choline positron emission tomography (PET)/CT scans.

#### Age-related changes

**Fig. 12:**
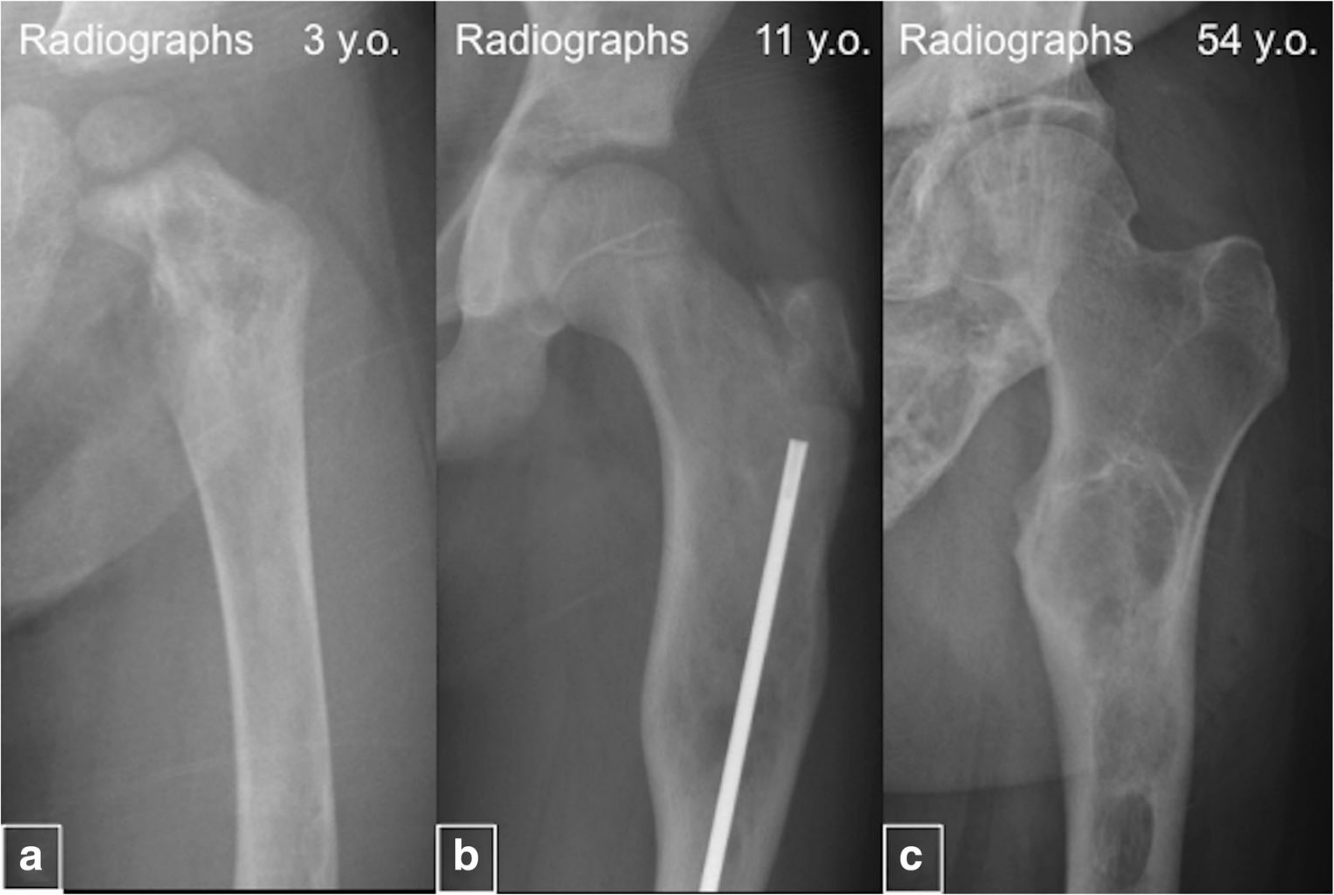
“Evolution” of the fibrous dysplasia (FD) lesions. **a** Radiograph of a 3-year-old demonstrates a typical heterogeneous-appearing FD lesion in the femur. **b** Radiograph from an 11-year-old demonstrates homogeneous and radiolucent FD lesion. **c** Image from a 54-year-old patient shows sclerotic FD lesions

**Fig. 13:**
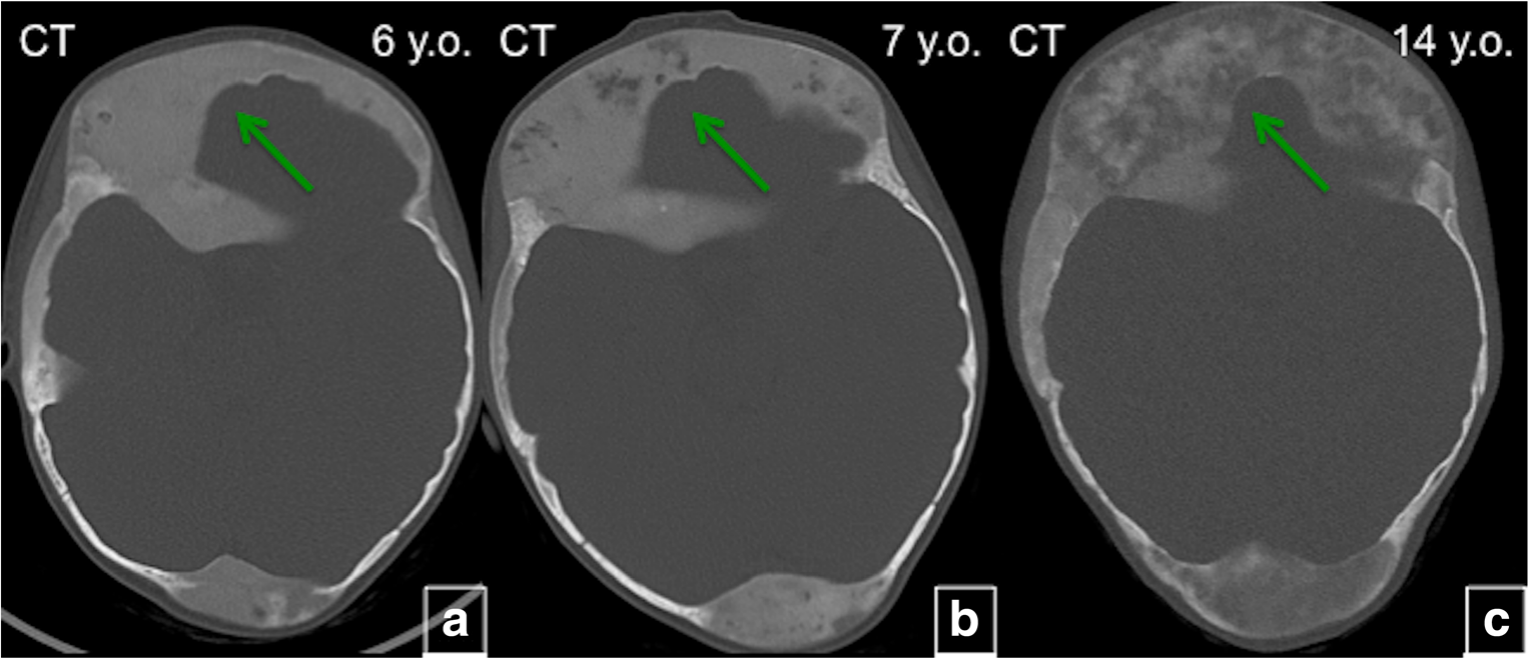
Age-related changes in fibrous dysplasia (FD). CT of the head on the same patient at age of 6 (**a**), 7 (**b**) and 14 years (**c**). Diffuse FD involvement with homogenous “ground glass” appearance (*green arrows*), which demonstrates the tendency to develop cystic lesions and become more heterogeneous with time (*green arrows*)

In children younger than 2 years, FD lesions in the appendicular skeleton often appear heterogeneous on radiographs and lack the classic ground glass appearance [11]. With time, mesenchymal cells that carry *GNAS* mutations undergo apoptosis, leading to a decreased number of FD cells and, thus, changing the classic radiographical appearance of ground glass to a more dense and sclerotic pattern (Fig. 12) [5]. Craniofacial lesions in older individuals typically become less homogeneous on CT, developing discrete radiolucent, cystic-appearing areas (Fig. 13). Rapid expansion of lesions is concerning for possible malignant transformation or ABC development.

#### Treatment-related changes

**Fig. 14:**
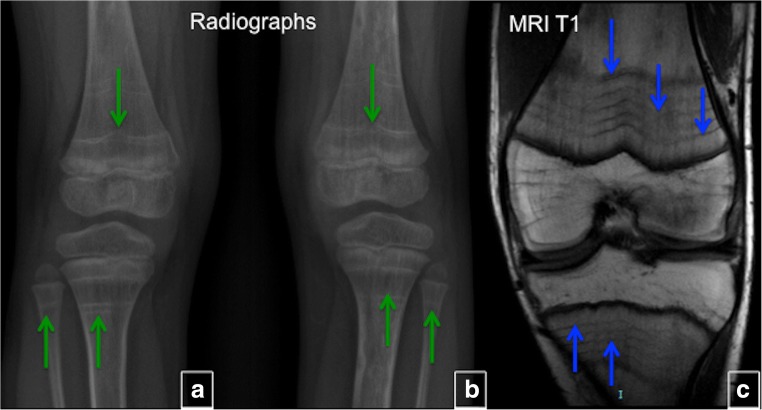
Bisphosphonate-induced lines. Administration of bisphosphonates results in the development of the parallel sclerotic metaphysial bands, which can be seen on radiographs (*green arrows*) and T1-weighted MRI (*blue arrows*)

Bisphosphonates, which are used in the treatment of FD-related osseous pain, change the appearance of the bone and are responsible for the development of parallel sclerotic metaphyseal bands. These bands develop in any growing child treated with bisphosphonates, and are not specific to FD [12]. Histologically, these bands are composed of horizontally arranged trabeculae containing calcified cartilage (Fig. 14).

## Complications and clinical course

The course and spectrum of FD complications is primarily determined by the disease location and burden. Many FD patients suffer quality of life impairment, which is increased in patients with greater disease burden and more pronounced in patients with polyostotic FD and MAS, secondary to their greater risk of developing complications such as deformities or fractures. About 92% of subjects who ultimately lose independent ambulation receive ambulatory aids prior to age 17. Pain and deformities, as well as benign and malignant bony matrix transformations, may occur in FD of any location. Depending on the lesion location, the disease may have different presentations and complications.

### Pain

Pain occurs in about two out of three patients with FD, is more prevalent and severe in adults than in children, and can significantly impact mobility and quality of life [13]. Interestingly, there is no association between the extent or location of disease and the likelihood of having pain; patients with mild FD may have debilitating pain, and patients with extensive disease may be pain-free. Patients with new onset of pain should undergo clinical and radiological evaluation for underlying metabolic, functional and orthopaedic complications or possible malignant transformation.

### Fractures

**Fig. 15:**
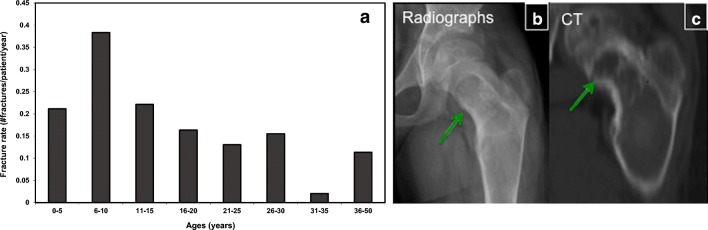
Fractures in fibrous dysplasia (FD). **a** Fractures are more frequent in childhood, with the highest rate occurring between 6 and 10 years of age. **b**, **c** Radiograph and CT of the left femoral bone demonstrate a fracture in the medial proximal femur in the settings of FD (*green arrows*) (reprinted from Dumitrescu and Collins [35])

Fractures are common and are most prevalent between the ages of 6 and 10 years, declining thereafter (Fig. 15) [14]. Several factors may predispose patients to fractures. One of them is hyperthyroidism that can cause clinically significant bone mineral loss through the direct stimulation of bone resorbtion. Moreover, abnormal FD cells secrete the protein fibroblast growth factor 23 (FGF-23), which is responsible for hypophosphatemic rickets and, therefore, increased osteomalacia and fracture rates. Fracture rates increase with higher disease burden and FGF-23 levels.

### Benign matrix transformation

**Fig. 16:**
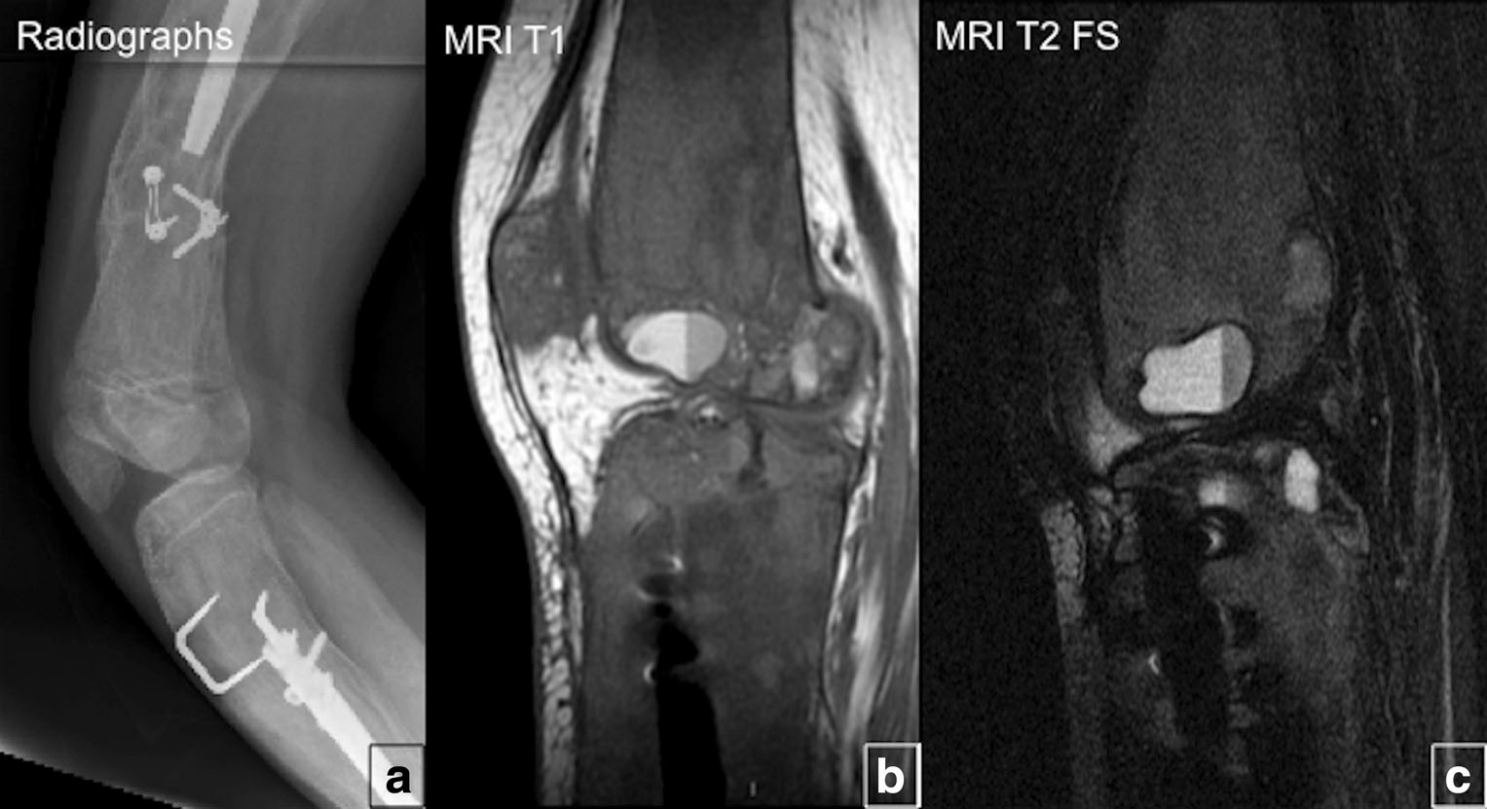
Benign fibrous dysplasia (FD) bone matrix transformation to aneurysmal bone cysts (ABCs). **a** Radiographs of the distal femur and the proximal fibula show heterogeneous FD lesions with surgical hardware. T1-weighted MRI (**b**) and T2 fat-suppressed images MRI (**c**) demonstrate ABCs

**Fig. 17:**
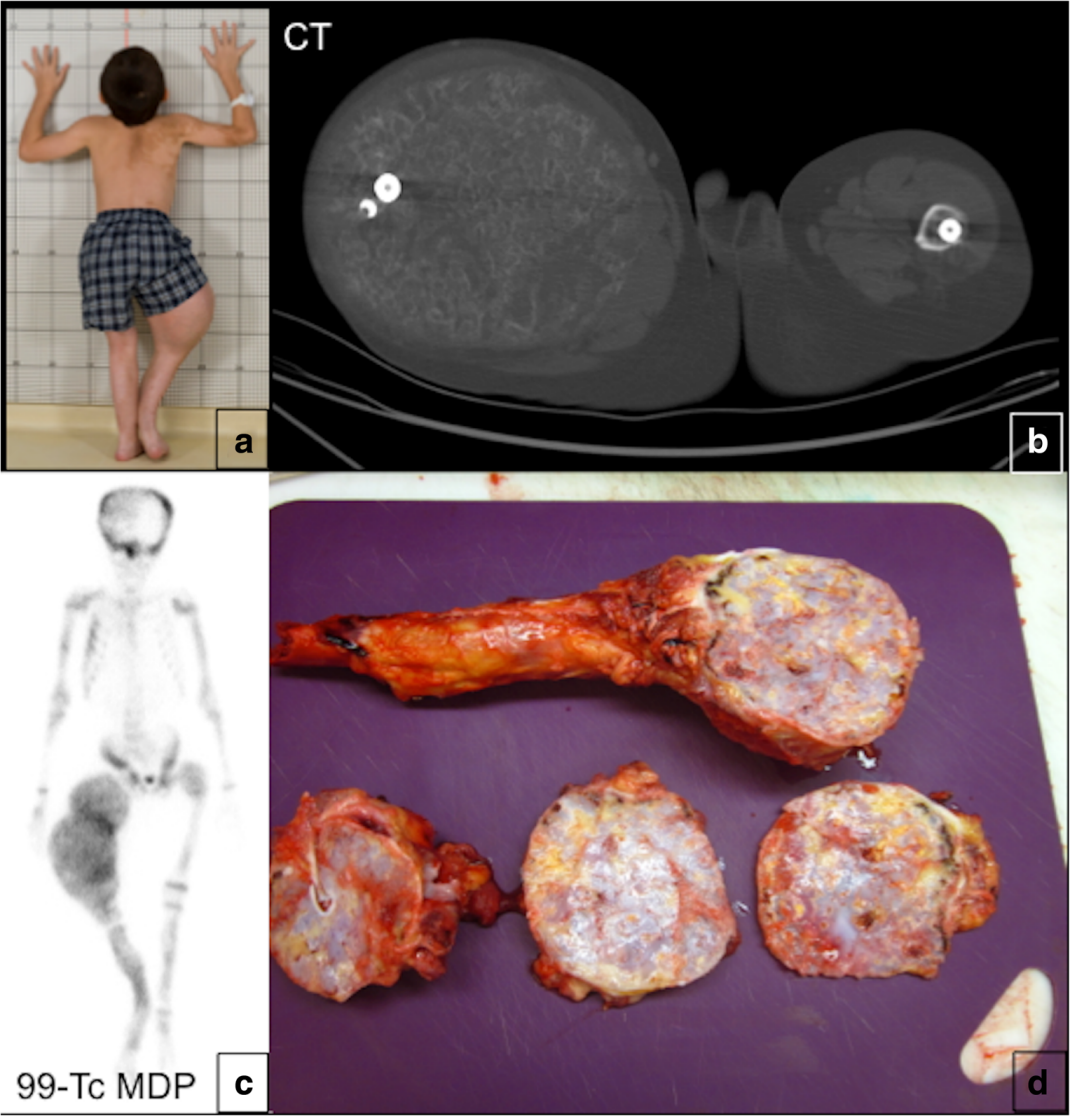
Benign myxoid bone matrix transformation in fibrous dysplasia (FD). **a** A patient with known FD of the femurs presents with an enlarging right thigh mass. **b** Axial unenhanced CT of the lower extremities shows a large heterogeneous mass in the right thigh replacing femur and causing mass effect on thigh muscles. Please note a normal position of the intramedullary road in the left femur. **c** The mass in the right thigh shows a focal 99m-Tc MDP radiotracer uptake. **d** Subsequently, the patient developed the same complication in the left leg. The image demonstrates extensive myxoid degeneration of the left femur

Benign secondary changes include aneurysmal bone cyst-like changes and myxoid changes (Figs. 16 and 17). ABC development is reported most often in the skull within pre-existing areas of FD in about 5% of patients. When an ABC forms in an FD bone, which is already soft and dysplastic, the cyst typically expands much more rapidly than FD would, leading to increased bone pain, fracture, progressive deformity, pathological fracture and neurological symptoms.

### Malignant matrix transformation

**Fig. 18:**
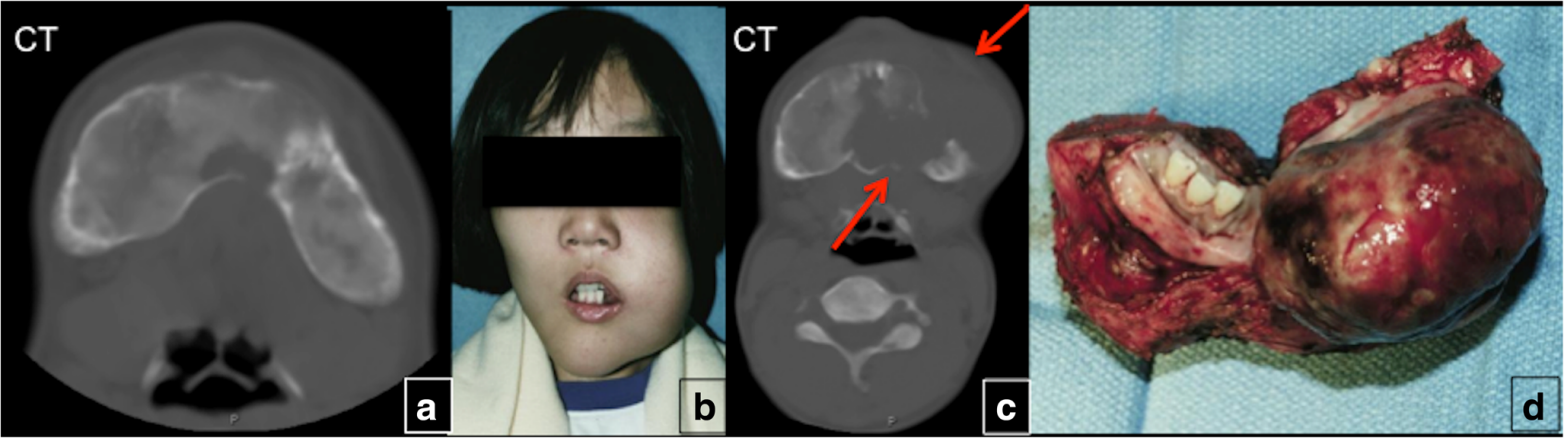
Malignant transformation of the fibrous dysplasia (FD) lesion. A patient with known craniofacial FD (**a**) presented with enlarging left jaw mass (**b**). CT of the facial bones demonstrated an aggressive lytic lesion with soft tissue component involving the left aspect of the mandible (*red arrows*) (**c**). The pathology showed malignant transformation of the mandibular FD lesion

Malignant transformation of FD lesions is a rare complication, occurring in up to 2.5% of cases. Malignant changes to osteosarcoma, fibrosarcoma, chondrosarcoma and malignant fibrohistiocytoma have been reported. Risk factors include concomitant growth hormone excess and a history of prior radiation treatment. Worsening pain and local swelling are suspicious clinical findings. Cortical destruction, osteolysis, adjacent soft tissue mass and the development of pathological fractures may suggest the development of low-grade central osteosarcoma or malignant transformation (Fig. 18) [15]. Making the diagnosis may be difficult, especially in cases of low-grade osteosarcoma, which shares similar histopathological features with FD. Immunohistochemical analysis for the expression of MDM2 and CDK4 proteins may assist in the tissue diagnosis, as malignancies will often express MDM2 or CDK4, while FD will not [16]. The treatment is based on management of the malignancy, and resection with adequate margins is necessary. In a group of 112 patients with FD who presented with acromegaly, six patients developed sarcoma of the skull base (5.4%). Three of these six patients had undergone pituitary irradiation 4–5 years previously. It is unclear if the malignant transformation was due to radiation exposure, secondary hormone factors or a combination [17].

### Craniofacial FD

**Fig. 19:**
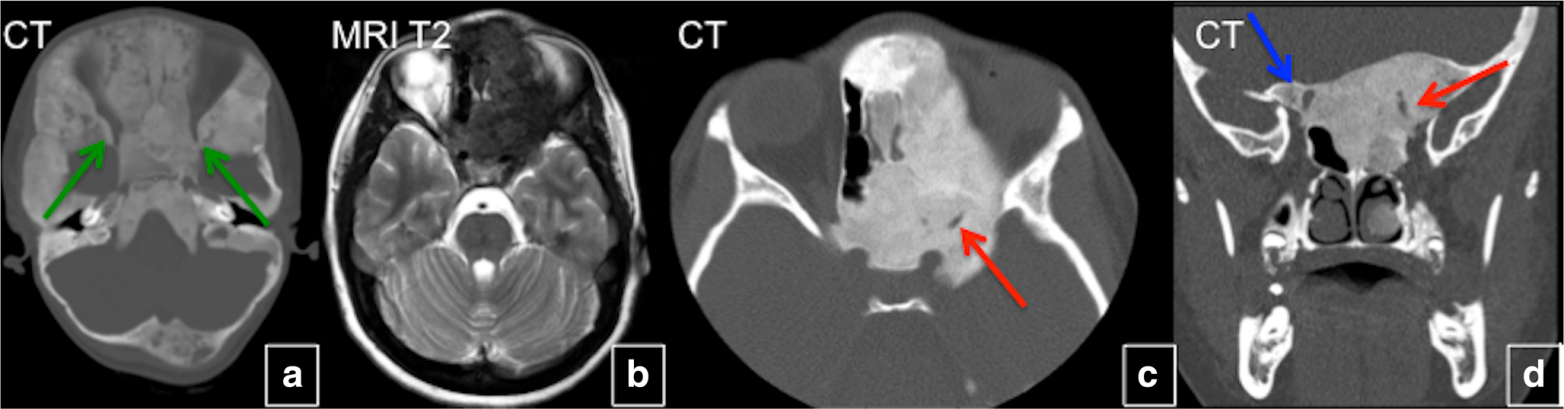
Optic nerves in craniofacial fibrous dysplasia (FD) in two different patients. **a** Extensive FD involving most of the facial bones and skull. Optic canals are narrowed but patent. **b**–**d** Expansile bone lesions in the left frontal bone, left sphenoid bones, ethmoid bone, and body of the sphenoid bone with marked narrowing and deformity of the left optic canal, causing left-sided blindness (*red arrows*). The right optic canal is narrowed (*blue arrows*). Vision in the right eye is preserved

**Fig. 20:**
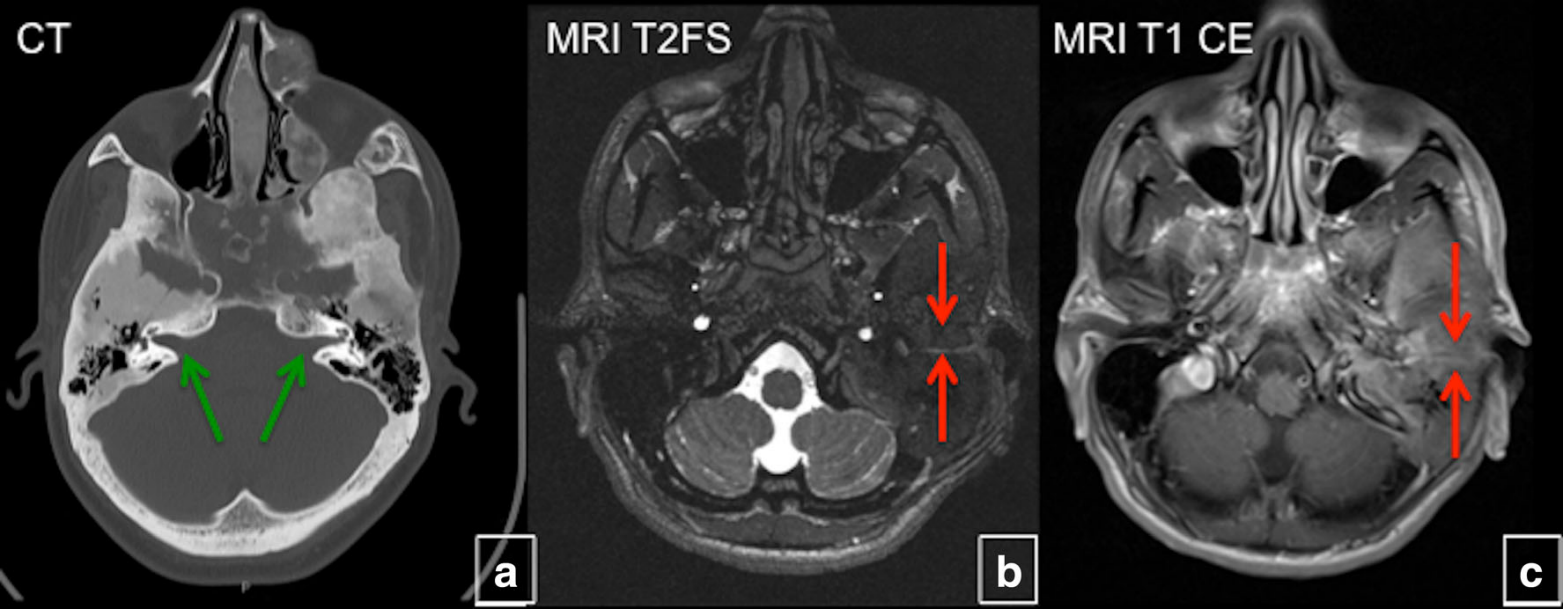
Evaluation of hearing loss in craniofacial fibrous dysplasia (FD). **a** Extensive fibrous dysplasia (FD) involving petrous bones bilaterally; however, the internal auditory canals are patent (*green arrows*). T2 fat-saturated (**b**) and T1 contrast-enhanced (**c**) MRI demonstrate a large FD lesion centred in the left petrous temporal bone with near complete obliteration of left external auditory canal (*blue arrow*), which contributes to patient’s hearing loss (*green arrow*)

**Fig. 21:**
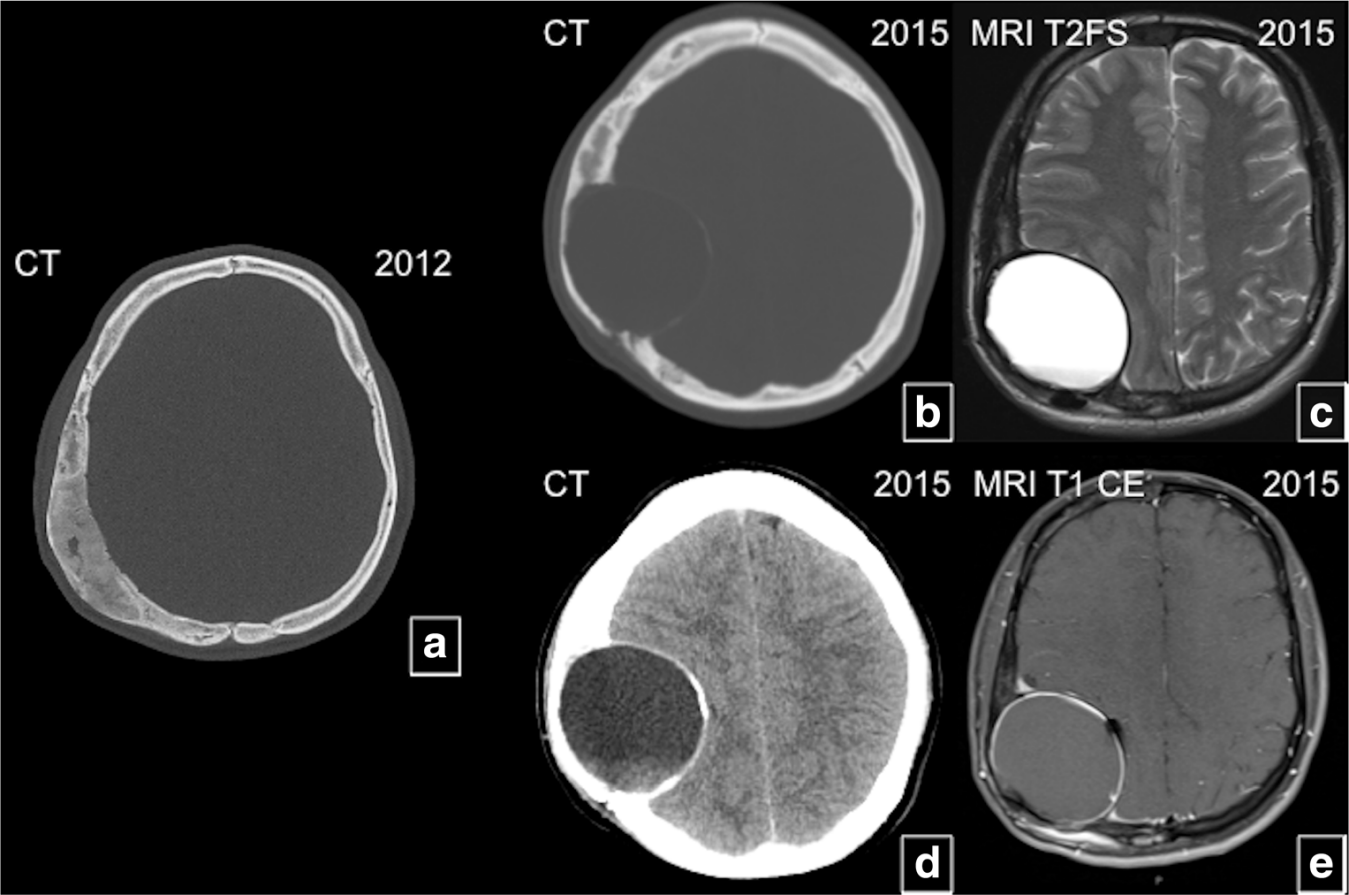
Brain compression in craniofacial fibrous dysplasia (FD). A patient with known right parietal bone FD (**a**) develops an aneurysmal bone cyst causing mass effect on adjacent brain (**b**–**e**)

Craniofacial bones are the most common FD location. The typical clinical presentation of craniofacial FD is a gradual, painless enlargement of the craniofacial region, leading to facial asymmetry. Rarely, deformities may lead to devastating functional and aesthetic consequences for affected individuals. Lesions in this location are not typically well demarcated, may cross sutures, and most commonly affect the zygomatico-maxillary complex and sphenoid bones. From late childhood to puberty, indolent growing mass lesions cause facial deformity and distortion of adjacent structures such as the optic and vestibulocochlear nerves, eye/globe, nasal airway, middle ear ossicles and teeth. Rapid enlargement of FD lesions in children and continued active disease with symptoms in adulthood is uncommon but may occur. Rare neurological complications include optic and vestibulocochlear nerve compression, scoliosis with spinal cord compression, and brain compression. Involvement of the orbit is common in FD (Fig. 19). Proptosis, globe dystopia, and hypertelorism may be seen on imaging. Any FD lesion surrounding the optic nerve and orbit should be reported, and a comprehensive neuro-ophthalmologic examination recommended. Optic nerve encasement is common and usually asymptomatic. Vision loss is uncommon, seen in only about 5% of patients and is frequently associated with growth hormone excess. More than 70% of patients with craniofacial FD have temporal bone involvement; however, the majority of them—more than 85% of patients in one study—have normal or near-normal hearing. Among the patients with temporal bone FD, hearing loss was identified in 41 ears (22.4%) and was conductive in 27 (65.9%), sensorineural in 12 (29.3%) and mixed in 2 (4.9%). Hearing loss, which is typically mild, develops secondary to the narrowing of the external auditory canal and the fixation of the ossicles within the epitympanum [18] (Fig. 20). A rare, but potentially concerning, complication is the development of cholesteatomas, FD of the skull base may be associated with growth hormone excess. In rare cases ABCs developing in the settings of FD may exert mass effect on adjacent brain parenchyma (Fig. 21). Sinuses may also be affected in craniofacial FD; however, the incidence of sinusitis is not greater than in the general population. Craniofacial lesions may affect a patient’s dentition.

### FD of the spine

**Fig. 22:**
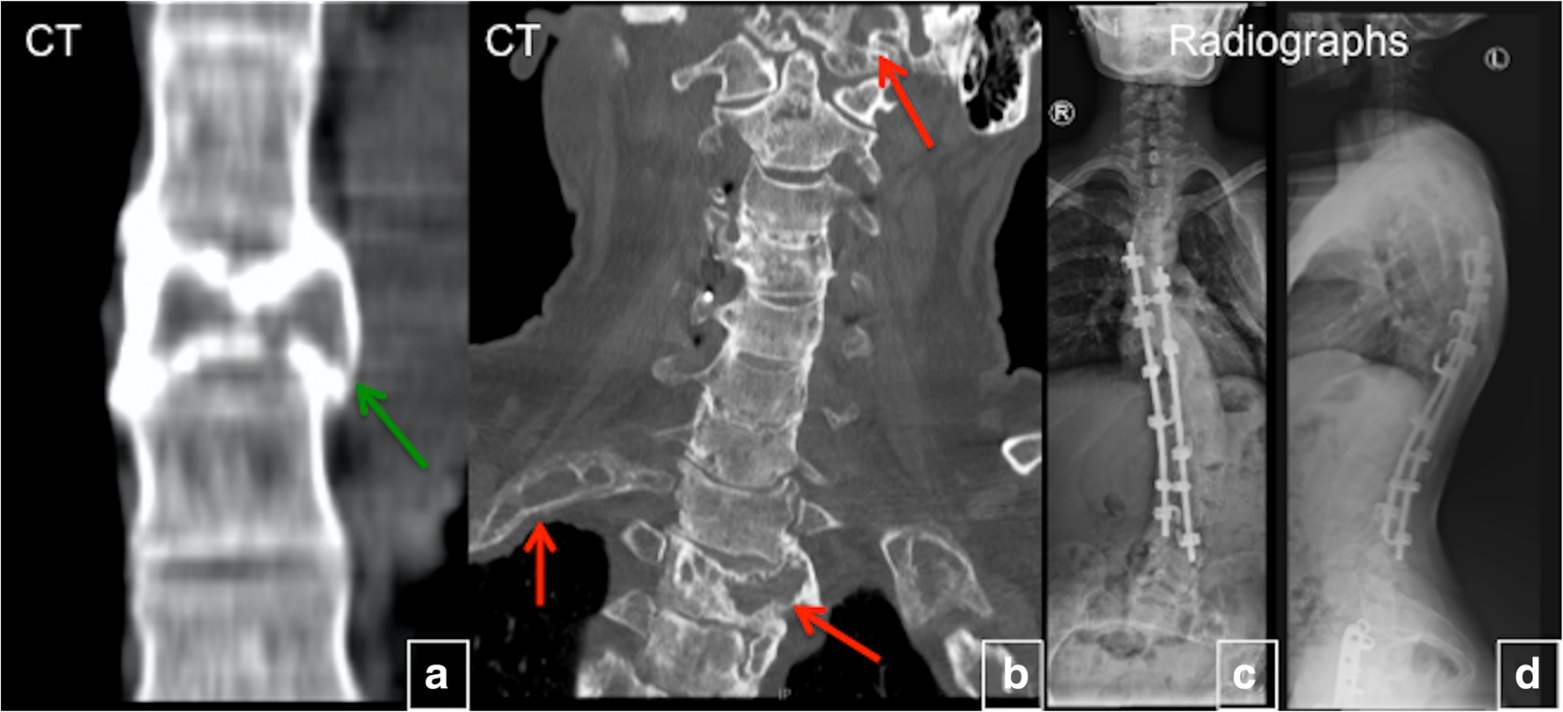
Fibrous dysplasia (FD) of the spine. **a** Monostotic FD of the T6 vertebral body complicated by a compression fracture (*green arrow*). **b** Polyostotic FD involving the spine, ribs and skull (*red arrows*). **c**, **d** Postsurgical radiographs after a spinal fusion for scoliosis in the settings of polyostotic FD

FD of the spine is very rarely observed in the absence of disease elsewhere in the body. The majority of spinal FD is the polyostotic form of the disease; the monostotic FD of the spine is exceedingly rare (Fig. 22) [19]. Uncomplicated monostotic lesions are generally asymptomatic and usually do not cause significant deformity. Patients with polyostotic FD may present with scoliosis, with a prevalence of 40–52%, which may be progressive, but back pain is uncommon. In rare cases, severe and progressive FD can lead to severe neurological complications, respiratory compromise and even death. Classic FD findings are not seen on plain radiographs of the spine but become visible with development of vertebral collapse or deformity. CT usually shows expansive lytic lesions with sclerotic rims and a decrease in vertebral body height. CT is also helpful in pre-surgical planning for detection of the degree of FD in each vertebral segment to be included in the fusion. MRI findings of patients with spinal FD are typically non-specific and similar to other locations. CT and MRI may demonstrate the extent of bony disease, paraspinal soft tissue extension, compromise of the spinal canal and spinal cord compression [20]. The diagnosis may be difficult, especially in adult patients with monostotic form, and may require biopsy. Spinal fusion is frequently effective and may be lifesaving.

### FD of long bones

**Fig. 23:**
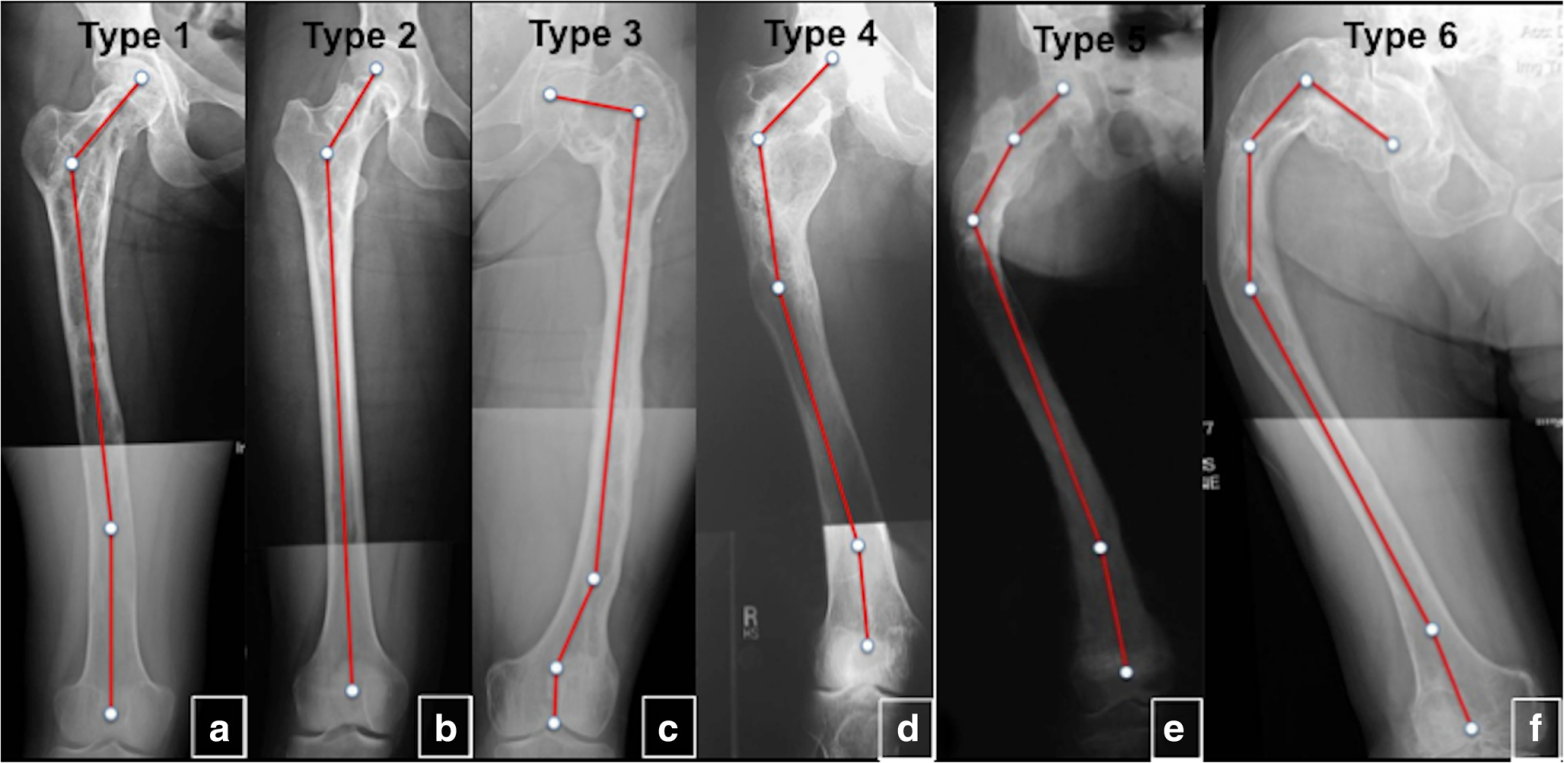
The classification of femur deformities in fibrous dysplasia (FD). **a** Type 1. The neck-shaft angle is within normal limits (135°), but a distal femur shows 16° valgus deformity. **b** Type 2. The neck-shaft angle is valgus (152°). **c** Type 3. The neck-shaft angle is varus (100°). A distal shaft 10° demonstrates varus deformity. Distal juxta-articular valgus deformity is also present. **d** Type 4. The neck-shaft angle is normal (125°). Proximal lateral (shepherd’s crook) and distal medial bowing of the femoral shaft are present. **e** Type 5. The neck-shaft angle is valgus (160°). Lateral bowing of the proximal femur (shepherd’s crook) and medial bowing of the distal femur are present. **f** Type 6. FD affects the entire femur. Lateral bowing of the proximal femur is present at two levels (shepherd’s crook) as well as medial bowing of the distal femur. The neck-shaft angle is varus (100°) (reprinted from Ippolito et al. [21])

The femur is the most common location of polyostotic FD (40%), leading to deformity, fracture and pain [21]. The risk of skeletal complications increases with a high skeletal burden, and in patients with MAS who have one or more endocrinopathies. Mechanical stress and repeated fractures result in progressive varus and bowing, leading to the classic shepherd’s crook deformity (coxa vara angulation of the proximal femur). Six patterns of deformity of the proximal part of the femur according to the neck-shaft angle measurement and the presence or absence of lateral bowing of the proximal femoral shaft were described (Fig. 23) [21].

### FD of the ribs

FD is the most common benign rib lesion. In adults, FD of the ribs is often discovered incidentally and is usually asymptomatic; however, it may present with obvious deformity or pain.

## McCune-Albright syndrome

Approximately 2–3% of patients with FD have extra-skeletal disease, known as McCune-Albright syndrome (MAS). Bone changes in MAS are often more severe than in polyostotic FD without extra-skeletal manifestations. MAS patients have the most extensive disease and the most complicated course of the disease, regularly experience multiple fractures, and require adequate surgical treatment [22].

### Ovarian cysts

**Fig. 24 a-d:**
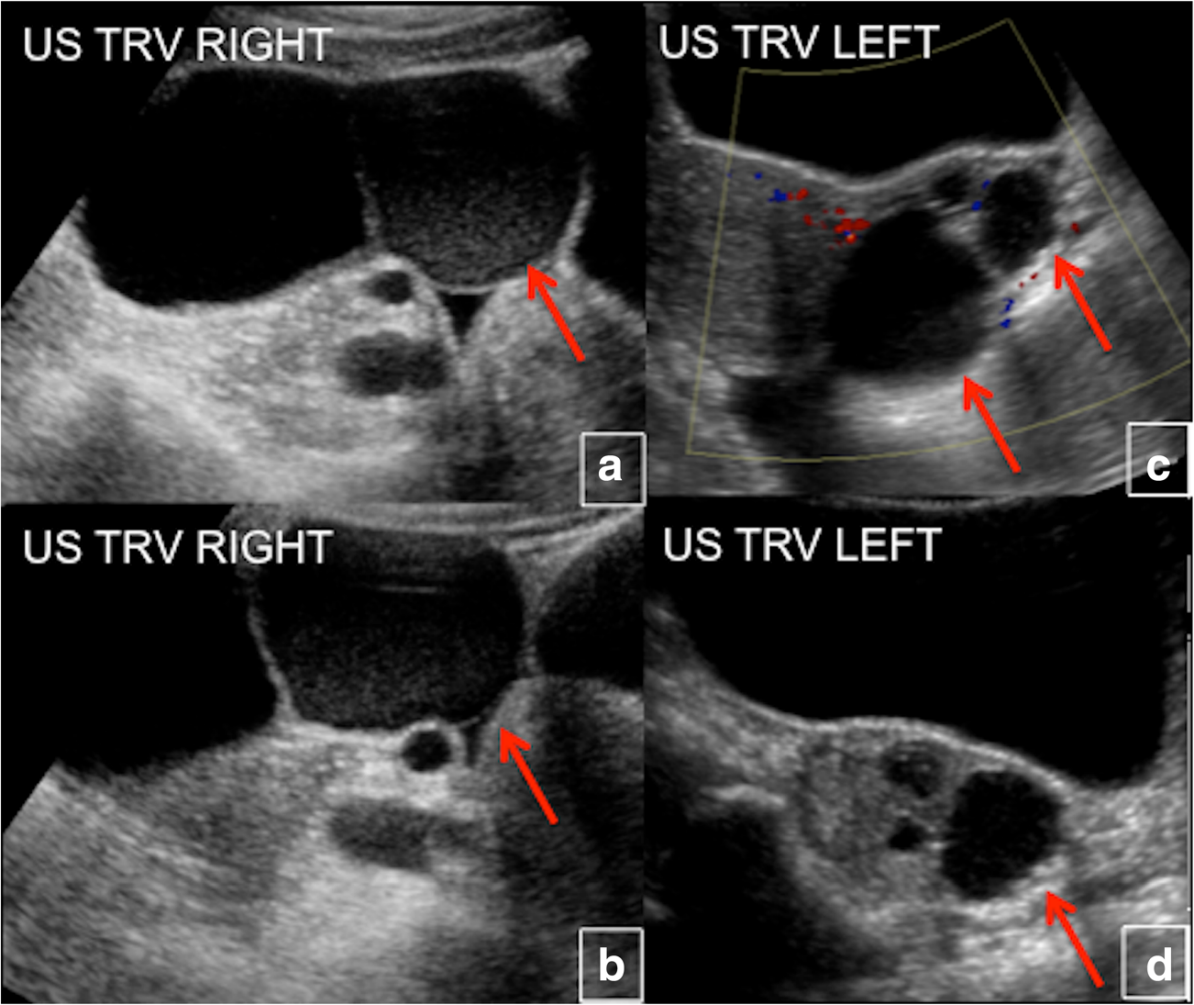
Bilateral ovarian cysts in McCune-Albright syndrome (*red arrows*)

Eighty-five percent of female patients have functionally active ovarian cysts, resulting in gonadotropin-independent precocious puberty. A typical ultrasound finding in these patients is a large unilateral ovarian cyst, which can sometimes be haemorrhagic and appear to have mixed cystic and solid elements (Fig. 24). Recurrent ovarian cysts lead to intermittent oestrogen production, resulting in breast development, growth acceleration and vaginal bleeding; during intervals between cyst formation, breast tissue typically regresses and oestrogen levels fall to prepubertal levels. Ovarian cysts typically continue into adulthood, leading to irregular menses. This has the potential to interrupt ovulatory cycles, which may increase the time to conception in adult women. Patients with MAS may have findings mimicking juvenile granulosa cell tumours, sometimes leading to unnecessary oophorectomies. Since the cysts are mostly gonadotropin-independent, it is common to see extreme asymmetry between the two sides. Ovarian torsion is a potential complication in women with large and persistent cysts, but it occurs rarely [23].

### Testicular abnormalities

**Fig. 25:**
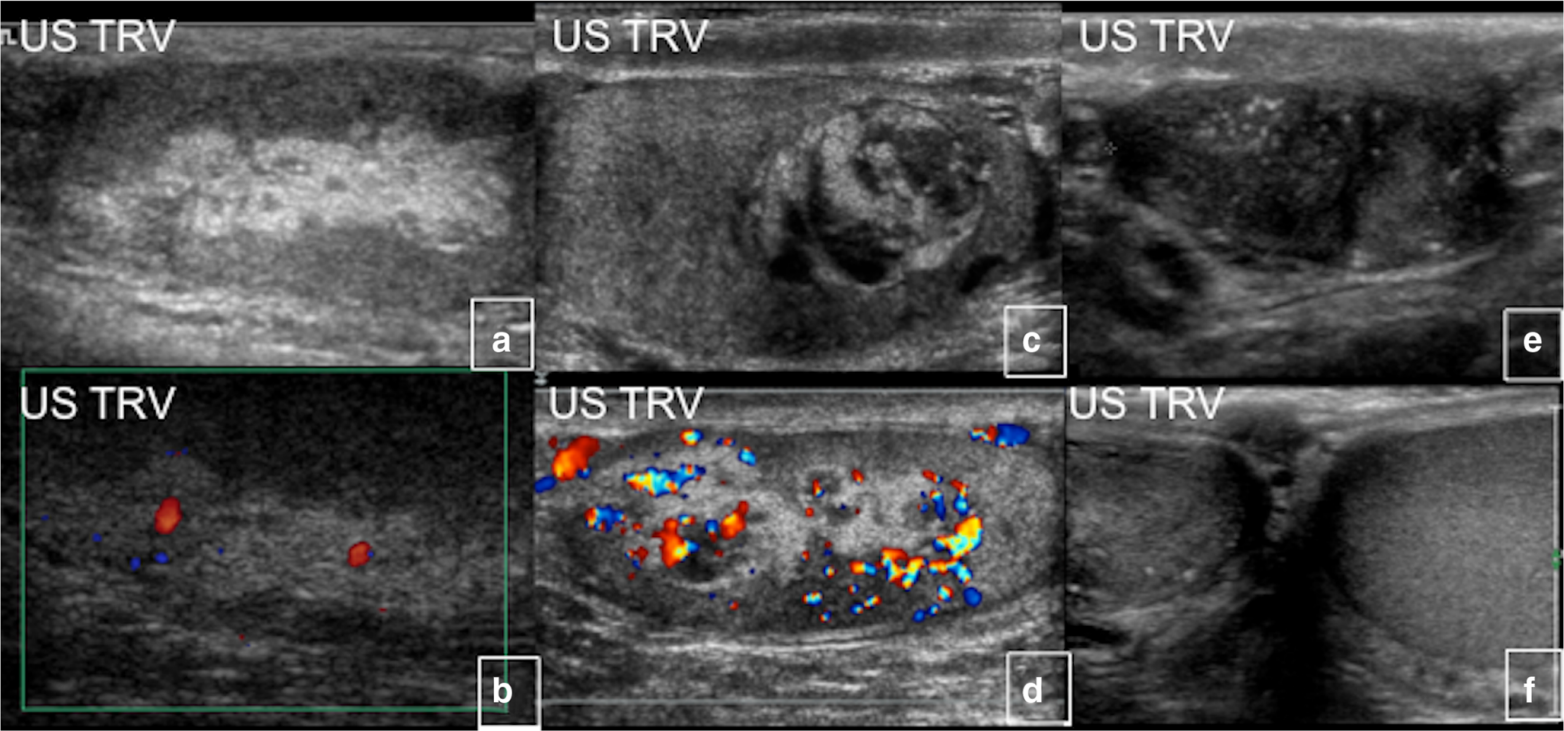
Testicular abnormalities in McCune-Albright syndrome in three different patients. **a**, **b** Ultrasound (US) of the testicles shows extensive echogenic material secondary to Leydig cell hyperplasia. **c**, **d** The heterogeneous appearance of the testicle with more focal, well-circumscribed areas of abnormal echogenicity. These imaging findings correlate with Leydig cell hyperplasia on pathology. **e**, **f** The right testicle appears atrophic and demonstrates inhomogeneous echotexure and multiple punctate calcifications

About 80% of male patients have testicular abnormalities that typically manifest as unilateral or bilateral testicular enlargement. Ultrasound may demonstrate hyperechoic lesions (49%), hypoechoic lesions (30%), microlithiasis (30%), heterogeneous lesions (47%) and focal calcifications (11%). These radiological findings often correspond to areas of Leydig and/or Sertoli cell hyperplasia. Imaging cannot differentiate benign from malignant pathology (Fig. 25). The malignant potential of testicular lesions is unknown, but it appears to be low. Precocious puberty is less common in males with MAS, occurring in 10–15% of patients with testicular abnormalities [24].

### Thyroid abnormalities

**Fig. 26:**
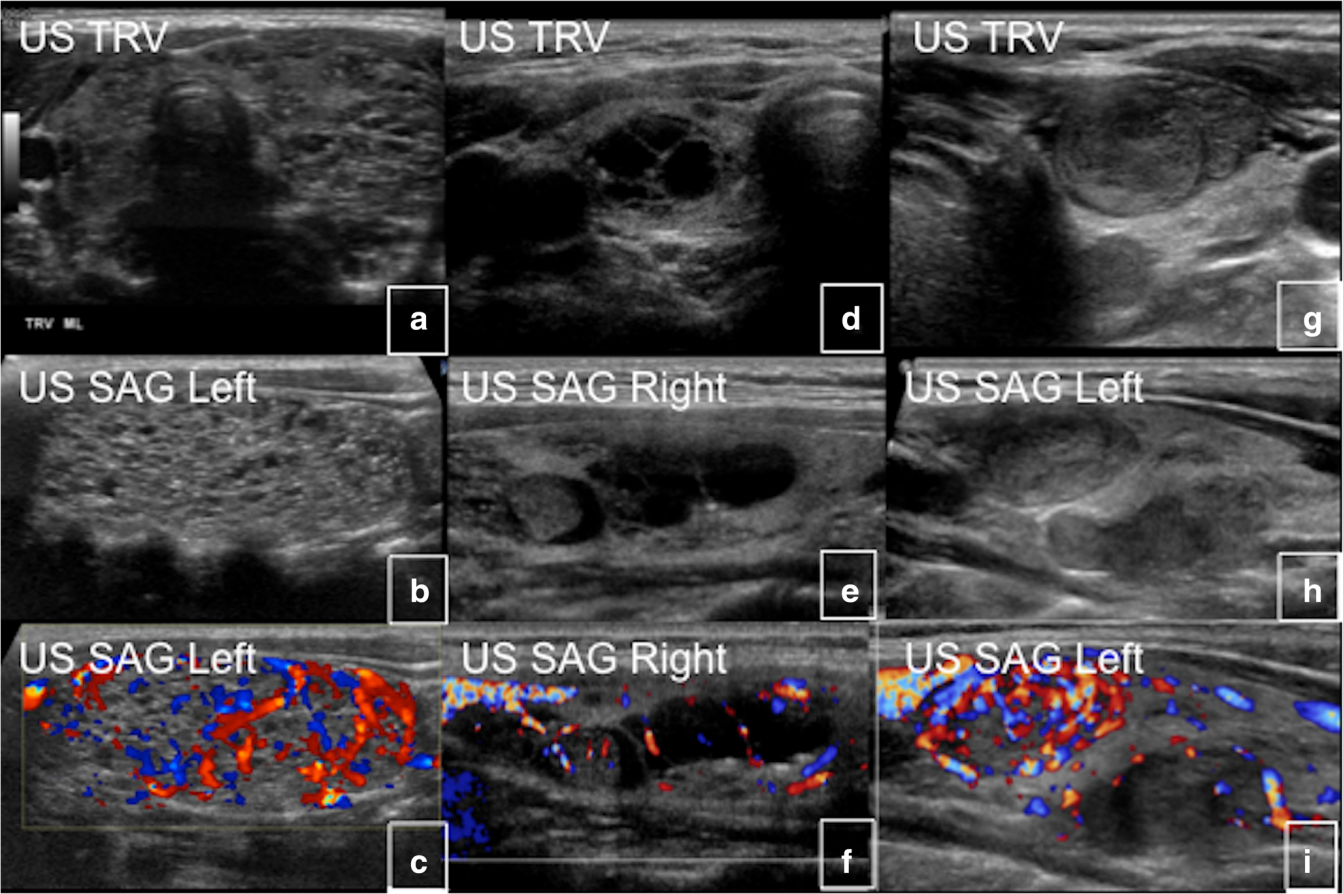
Thyroid abnormalities in McCune-Albright syndrome (MAS) in three different patients. **a**–**c** Ultrasound (US) of the thyroid gland shows typical microcystic changes. Macrocystic pattern (**d**–**f**) and solid thyroid nodules (**g**–**i**) can also be seen in patients with MAS

Hyperthyroidism is common (38%); evidence of thyroid involvement without frank hyperthyroidism is even more common (63%). Ultrasound typically demonstrates diffuse benign changes such as multinodular goitre, and mixed cystic and solid nodules. Malignant transformation of affected thyroid tissue has rarely been reported (Fig. 26) [25].

### Pituitary disorders

**Fig. 27:**
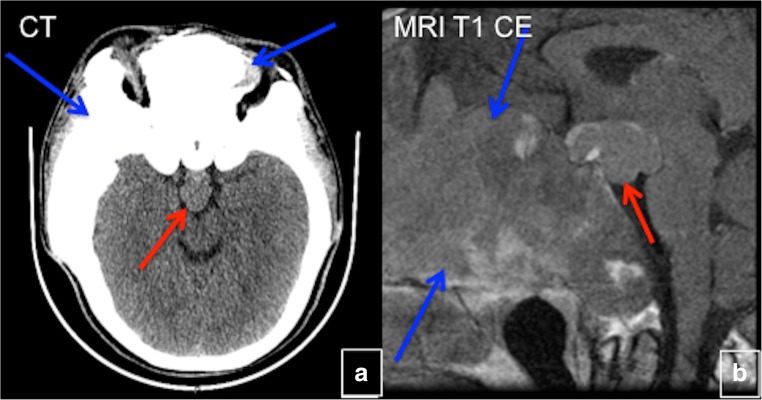
Pituitary adenoma in craniofacial fibrous dysplasia (FD). **a**, **b** A patient with craniofacial FD (*blue arrows*) with pituitary macroadenoma (*red arrows*) on CT (**a**) and T1 contrast-enhanced MRI (**b**)

Ten to fifteen percent of individuals with MAS harbour *GNAS* mutations in the anterior pituitary that can lead to autonomous growth hormone production, typically accompanied by hyperprolactinaemia. The abnormality is usually diagnosed during young adulthood and is almost always associated with skull base FD. Among patients with growth hormone excess, adenoma can be seen in about 54% and macroadenoma in more than two-thirds of those cases (Fig. 27). About 40% of acromegalic patients have diffuse somatotroph hyperplasia without adenoma, in which only diffusely abnormal pituitary enhancement is seen. In many cases of growth hormone excess, the pituitary appears normal on MRI. However, even when an adenoma is seen on imaging, the pituitary is still likely to be diffusely involved on a histological level in patients with growth hormone excess. Therefore, removal of an adenoma is unlikely to be curative [26].

### Gastrointestinal abnormalities

**Fig. 28:**
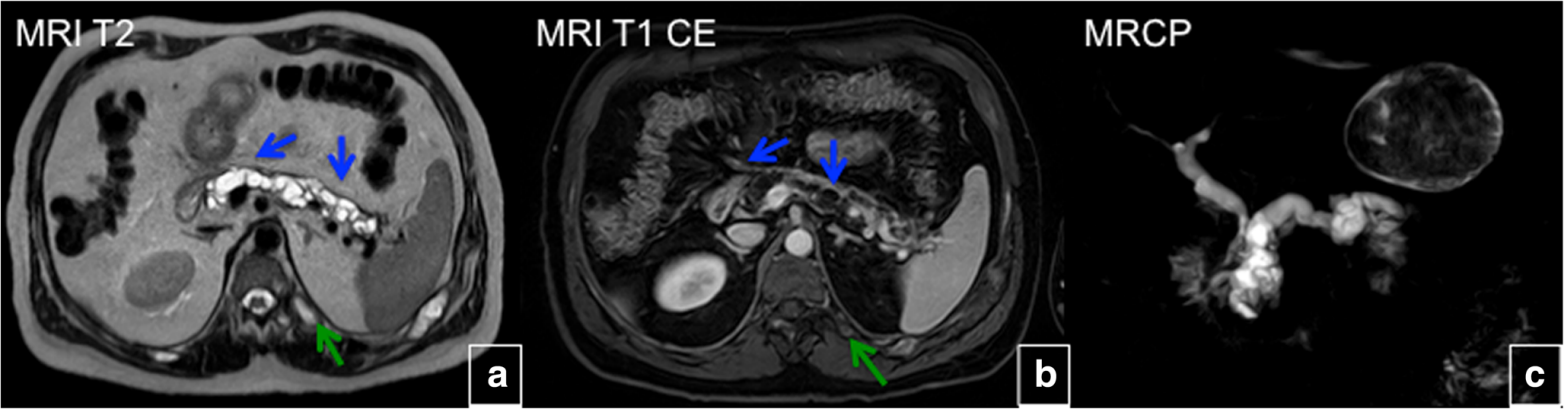
Intraductal papillary mucinous neoplasms (IPMNs) of the pancreas in McCune-Albright syndrome (*blue arrows*). T2-weighted MRI (**a**), T1 contrast-enhanced MRI (**b**). Coronal maximum intensity projection from a 3D T2-weighted MRCP acquisition shows IPMNs (**c**). Please note, a fibrous dysplasia lesion in the left rib (*green arrows*)

Fifteen percent of patients with MAS have activating *GNAS* mutations in the pancreas, resulting in intraductal papillary mucinous neoplasm (IPMN). IPMN can progress to invasive adenocarcinoma, suggesting that IPMN is an important precursor to pancreatic malignancy. IPMNs occur at an early age, and optimal care is evolving. Recently, liver adenomas and choledochal cysts were also described in patients with MAS. Screening symptomatic patients with abdominal MRI or magnetic resonance cholangiopancreatography (MRCP) is recommended (Fig. 28) [27, 28].

### Skin abnormalities

Café-au-lait spots on skin are one of the first manifestations of the disease and are typically described as having a “coast of Maine appearance”, with jagged borders distributed along the midline of the body.

## Mazabraud syndrome

Patients with Mazabraud syndrome have FD lesions and myxomas, typically located in the vicinity of the bone lesions. Mazabraud syndrome is rare. Until now, fewer than 100 cases have been reported worldwide. The syndrome is more common with the polyostotic form of FD.

### Intramuscular myxomas

**Fig. 29:**
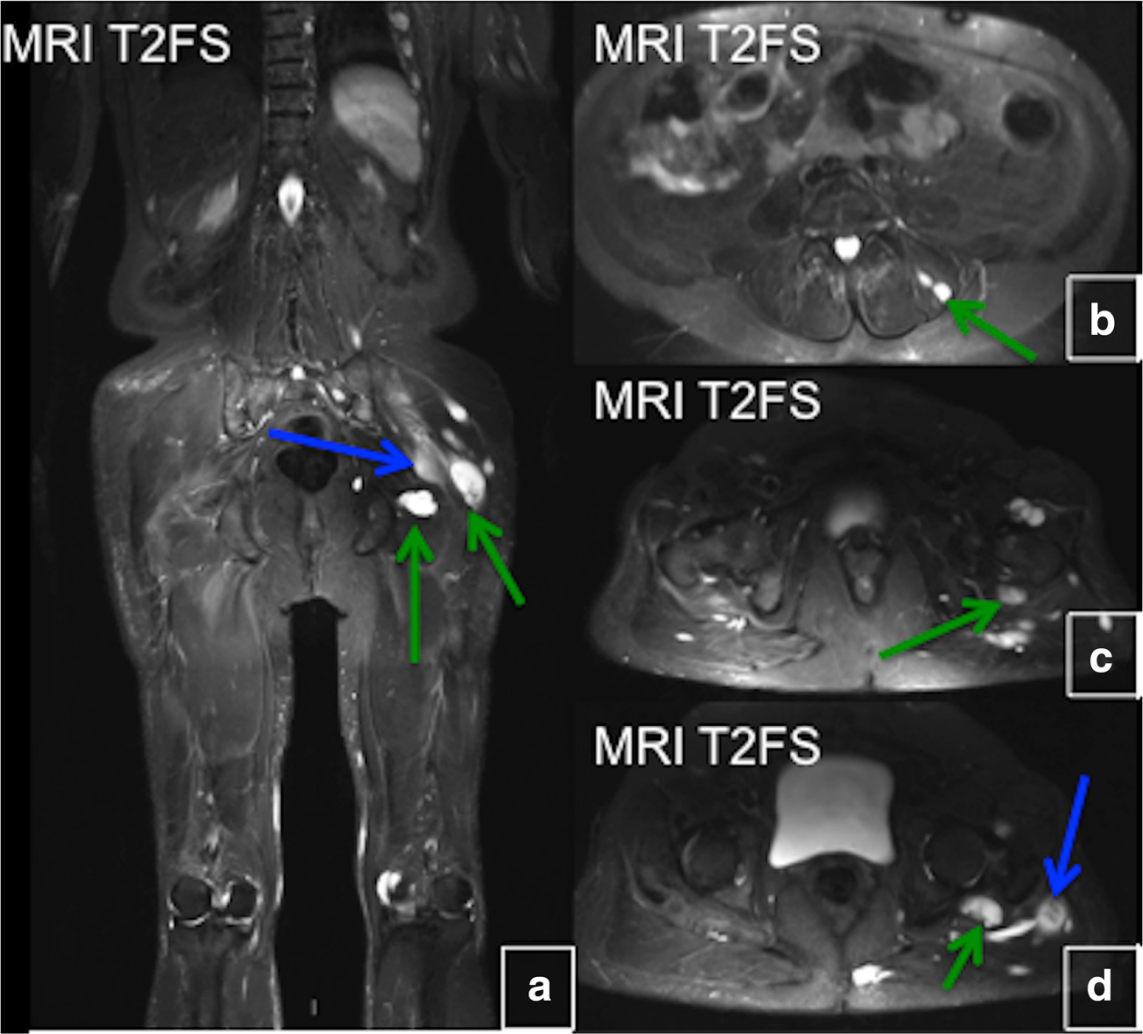
**a**–**d** Intramuscular myxomas in Mazabraud syndrome. Sagittal and axial T2-weighted fat-saturated magnetic resonance images demonstrate several high-signal-intensity lesions (*green arrows*). Some of the lesions show peripheral oedema (*blue arrows*)

Myxomas may be single or multiple. In general, the onset of FD predates the appearance of intramuscular myxomas, and soft tissue lesions become apparent many years later—usually in the 5th or 6th decade of life. The disease is more frequent in women than men, and patients are often asymptomatic. On imaging, intramuscular myxomas appear as a round or oval-shaped intramuscular soft-tissue mass in close proximity to bony lesions. The most common myxoma location is the thigh with an associated FD lesion in the femur. Sometimes myxomas may be seen in upper extremities, calves and buttocks. Due to high water content, they appear hypodense on CT and show minimal surrounding fat atrophy and mild surrounding oedema. On MRI, myxomas demonstrate hypointense T1 and hyperintense T2 signal (Fig. 29). Typically, lesions show no 18-F-FDG avidity on PET/CT. Biopsy is not recommended. Myxomas are benign, and conservative management is indicated. They can be excised if symptomatic, and simple local excision is sufficient in most cases; however, they frequently recur after surgical resection. No malignant transformations of myxoma have been reported, but six cases of malignant transformation of FD lesions into oestrogenic sarcoma in patients with Mazabraud syndrome have been reported, justifying the recommendation of clinical follow-up [29].

## Management, treatment and follow-up

**Fig. 30:**
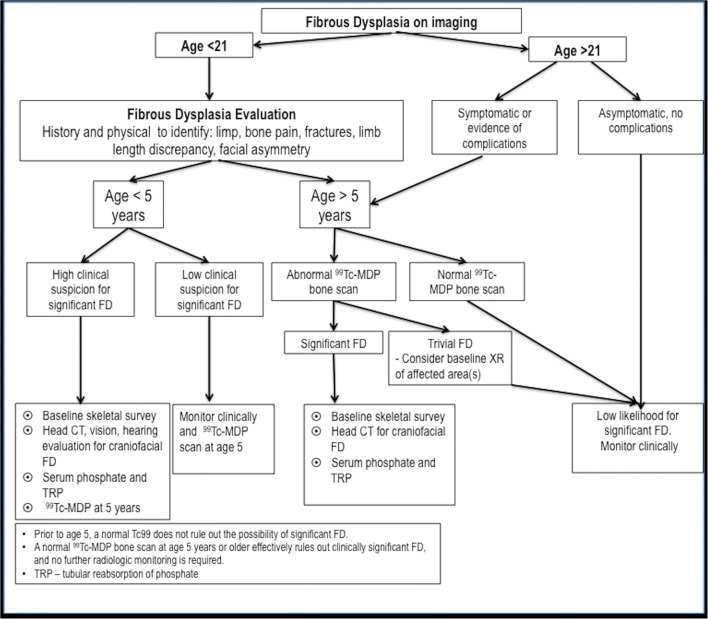
An algorithm of the management of fibrous dysplasia

Clinical management of new cases of FD is complex (Fig. 30). The key to management is to first establish the extent of the disease. Before the age of 5 years, a bone scan may not show all areas that will ultimately be involved with FD. Small foci of FD may not be detected by the bone scan. After age 6 years, all affected areas of FD are usually detectable. All young patients with FD and older patients with the polyostotic form of the disease, even in the absence of any history or clinical findings suggestive of endocrine dysfunction, should be evaluated by an endocrinologist. Patients with craniofacial FD require an evaluation by a craniofacial specialist. In cases of polyostotic or extra-skeletal disease, FD that has a typical radiological appearance does not generally require tissue diagnosis. Biopsy is indicated for histological confirmation only in cases that do not present a typical radiographic appearance, or in cases of isolated monostotic lesions.

There are no medications capable of altering the disease course; therefore, the treatment of the disease is mainly palliative, focusing on optimising function and minimising morbidity related to deformities and fractures. Antiresorptive therapy with bisphosphonates has been advocated due to high levels of bone resorbtion frequently seen in FD tissue; while these medications may be helpful for FD-related bone pain, there is no evidence that they improve bone quality or a radiographic appearance of FD lesions. Treatment of monostotic FD is dependent on symptom management and clinical presentation. The typical asymptomatic lesion, which is identified incidentally, requires the follow-up with serial radiographs to confirm that the lesion is biologically inactive and mechanically insignificant. Symptomatic or atypical lesions may require surgical excision for histological confirmation. Surgical interventions for pain relief are rarely effective. When surgical interventions are indicated, monostotic lesions are typically treated with conventional surgical procedures.

Management of patients with polyostotic FD is very challenging, as a majority of these patients are growing children. Radiographs are used selectively to monitor the progression of lesions initially identified using the bone scans. In the absence of fractures or symptoms, the follow-up for a child with FD consists of twice-yearly clinical evaluations with special attention to limited range of motion, obvious angular deformity, and a limb length discrepancy. The appendicular skeleton can often be evaluated without radiographs, with the exception of the proximal femur. Deformity in the femur may be progressive with little visible deformity until the angulation is severe; therefore, serial radiographs should be obtained.

The craniofacial form of the disease is the most common type of FD and the most difficult form to manage. “Clinical guidelines for the management of craniofacial fibrous dysplasia”, which were published in 2012, provide detailed and comprehensive recommendations [4]. In the case of a quiescent FD lesion in which the patient does not complain of facial deformity, observation and monitoring for changes is an acceptable treatment modality [4]. An annual CT may be necessary for the first 2 years after establishing the diagnosis; however, the interval may be lengthened based on the clinical findings [4]. Patients with aggressive and rapidly expanding FD or new onset pain or paraesthesia/anaesthesia require immediate evaluation by a maxillofacial surgeon, ENT or craniofacial surgeon and CT imaging [30]. Patients with acute visual changes or vision loss should undergo CT of the cranial base and immediate referral to a neurosurgeon or craniofacial surgeon, as well as to a neuro-ophthalmologist. Prophylactic surgical optic nerve decompression increases the risk of vision loss and is not recommended [4]. Pituitary MRI should be recommended for patients who have biochemical evidence of growth hormone excess [4].

Patients with scoliosis may be evaluated by clinical exam alone; however, for patients with progressive worsening deformity, radiographs of the spine are appropriate. Progressive scoliosis often responds favourably to surgical instrumentation.

Patients with upper extremity fractures may be treated conservatively, while fractures of weight-bearing bones are often managed surgically, preferably with intramedullary devices. On follow-up radiographs, radiologists should be able to detect any residual angulation, as remodelling and correction of residual angulation does not typically occur as quickly and as reliably in FD as it would in normal bone. Moreover, bone grafting is frequently ineffective [31].

In all cases where benign and malignant bone matrix transformation is suspected, patients should undergo CT and MRI. Surgical management is required in all cases of benign and malignant bone transformation.

Management of extra-skeletal abnormalities in patients with MAS and Mazabraud syndrome is mostly conservative, but follow-up imaging might be recommended (Table 2). Patients with FD have a more than threefold increased risk of developing breast cancer at a younger age compared with the general population, particularly in women with the more severe forms of FD [32].

**Table 2:** Suggested follow-up imaging for patients with fibrous dysplasia, McCune-Albright syndrome and Mazabraud syndrome (an expert opinion, based on the NIH cohort)

Involvement	Organs involved	Frequency of involvement^a^	Clinical problem	Suggested radiological follow-up
Bone lesions	All lesions	100%	Fractures, benign and malignant matrix transformation	Initial bone scan to assess the extension of disease. CT of the affected area/bones to evaluate changes in pain, rapid enlargement, local changes.
	Craniofacial bones	80%	Vision/Hearing	Head CT at baseline. Repeat periodically in childhood to monitor progression. Repeat as needed for symptomatic lesions in adulthood.
	Femur	91%	Deformities	Measure neck-shaft angle to identify progressive femoral neck deformation on X-ray.
	Axial skeleton	63%	Scoliosis	Closely monitor for scoliosis on X-ray; surgical fixation if Cobb angle > 50 degrees.
Extra-skeletal	Thyroid	66%	Hyperthyroidism (38%), autoimmune thyroiditis, thyroid cancer (1.3%)	Thyroid ultrasound at baseline and periodically to follow abnormalities.
	Pituitary	10–15%	Adenoma, hyperplasia without adenoma	MRI brain at baseline for patients with abnormal pituitary function.
	Testicles	85%	Macroorchidism, Leydig or Sertoli cell hyperplasia, testicular germ cell tumour	Testicular ultrasound at baseline and periodically to follow abnormalities.
	Ovaries	85%	Autonomous ovarian cysts	Pelvic US if breast development, vaginal bleeding or signs of estrogenisation below age 6–7 years.
	GI tract	32%	Pancreatic IPMN	MRI of abdomen with MRCP follow-up in 6–12 months if IPMNs 10–20 mm; 6 months follow-up if > 20 mm or demonstrates suspicious features
	Intramuscular myxomas in Mazabraud syndrome	100%	Asymptomatic	No follow-up

^a^In the NIH cohort

## Radiation exposure in patients with fibrous dysplasia

Patients with FD undergo frequent imaging, thus are exposed to radiation at a far greater rate than typical patients. Although the potential role of radiation exposure related to imaging for diagnostic and follow-up purposes have not been evaluated, concern about radiation-induced malignancies has been raised. Although malignant transformation of FD occurs in a very small number of patients, it is still unclear whether malignant transformations develop secondary to radiation-induced carcinogenesis from imaging or because of the biology of the FD. Efforts should be made to reduce cumulative radiation risks for these patients and, if possible, optimise utilisation of non-ionising imaging alternatives [33].

## Conclusions

1. FD is a complex disease, and the knowledge of its unique pathogenesis involves not only bone, but also multiple other organs.
2. FD lesions are characterised by age-related histological, radiographic and clinical transformations.
3. Radiologists play a crucial role in the identification of osseous complications associated with FD.
4. The craniofacial form of the disease is the most common type of FD and the most difficult form to manage. It requires clinical and radiological evaluation and follow-up.
5. Patients with MAS may have different extra-skeletal abnormalities (ovarian cysts, testicular changes, pituitary adenoma or IPMN), which often require follow-up.
6. Many patients with FD undergo repeated imaging with radiation; therefore, high radiation exposure is a concern. Efforts should be made to reduce cumulative radiation risks for these patients.
